# Poly(ester imide)s with Low Linear Coefficients of Thermal Expansion and Low Water Uptake (VIII): Structure–Flame Retardancy Relationship

**DOI:** 10.3390/polym16141967

**Published:** 2024-07-10

**Authors:** Masatoshi Hasegawa, Yuta Takeuchi, Takayuki Saito

**Affiliations:** Department of Chemistry, Faculty of Science, Toho University, 2-2-1 Miyama, Funabashi, Chiba 274-8510, Japan

**Keywords:** poly(ester imide)s, polyimides, heat resistance, linear coefficients of thermal expansion (CTE), linear coefficients of humidity (hygroscopic) expansion (CHE), water uptake, elongation at break, film toughness, flame retardancy

## Abstract

A series of ester-linked tetracarboxylic dianhydrides containing multiple *para*-phenylene units (TA-*p*Phs) was synthesized to obtain novel modified polyimides, namely poly(ester imide)s (PEsIs). The flame retardancy and film toughness of PEsIs tended to deteriorate with the structural extension of the repeating units (or monomers) via ester groups. To identify the structural factors necessary for achieving the highest flame retardancy rank (UL-94, V-0), we systematically investigated the structure–property relationships of a series of TA-*p*Ph-based PEsIs. Among them, a PEsI derived from *para*-quaterphenylene-containing TA-*p*Ph (TA-DPQP) and *p*-phenylenediamine (*p*-PDA) exhibited the best property combination, featuring an extremely high glass transition temperature (*T*_g_), very low linear coefficient of thermal expansion (CTE), low water uptake (*W*_A_), ultralow linear coefficient of humidity (hygroscopic) expansion (CHE), unexpectedly high film toughness, and excellent flame retardancy (V-0 rank). Moreover, we examined the effects of substituents of TA-*p*Ph and discussed the mode of action for the increased film toughness. This study also investigated the structure–property relationship for a series of PEsIs derived from isomeric naphthalene-containing tetracarboxylic dianhydrides. Some of the PEsIs obtained in this study, such as the TA-DPQP/*p*-PDA system, hold promise as novel high-temperature dielectric substrates for use in flexible printed circuits.

## 1. Introduction

Wholly aromatic polyimides (PIs) are heat-resistant polymeric materials indispensable as electrical insulation films, owing to their resistance to a solder-reflow process at 260 °C for electrically connecting various electronic components to circuits [1,2,3,4,5,6,7,8,9]. The rapid progress in performance improvement, functionalization, thickness and weight reduction, and flexibilization of electronic devices has been supported by numerous studies on the chemistry, physics, properties, functionality, and practical applications of PIs as dielectric materials [10,11,12,13,14,15,16,17].

PIs have been mainly used as electrical insulation films for flexible printed circuits (FPCs), which can be mounted while freely folding. FPCs are generally fabricated via the photolithography-assisted formation of high-resolution copper circuits using prefabricated single-/double-sided copper clad laminates, in which a non-thermoplastic PI film (base film) is laminated with copper foils using conventional (typically, epoxy resin–rubber composites) or thermoplastic PI adhesives. Recently, FPCs have found wide use in electronic devices, such as automotive electrical parts, mobile phones, tablets, computers, hard disks, industrial robots, and medical equipment.

The KAPTON^®^ H film (DuPont, Wilmington, DE, USA) [18], obtained from pyromellitic dianhydride (PMDA) and 4,4′-oxydianiline (4,4′-ODA), is used as a typical base film in FPCs because of its outstanding chemical and physical heat resistance, excellent flame retardancy, reliable electrical insulation performance, resistance to various chemicals, and good mechanical properties. However, this PI film does not always meet the requirements associated with the recent rapid reduction in the circuit line pitch of FPCs. Therefore, the dimensional stability of FPC base films against repeated thermal cycling during device fabrication should be increased, specifically by reducing the linear coefficients of thermal expansion (CTEs) of PI base films. 

In view of the above, commercial low-CTE base films, such as the KAPTON^®^ EN film (DuPont, Wilmington, DE, USA) [19] and UPILEX^®^-S film (UBE, Japan) [20], are in high demand. However, even these low-CTE PI films do not always show sufficiently low water uptakes (*W*_A_s) and high dimensional stability against water/moisture absorption. Therefore, PIs exhibiting low CTEs, low *W*_A_s, and low linear coefficients of humidity (hygroscopic) expansion (CHEs) are highly sought after. However, it is difficult to satisfy these new requirements while preserving the advantages of the KAPTON^®^ H film, as long as existing monomers are used.

**Scheme 1:**
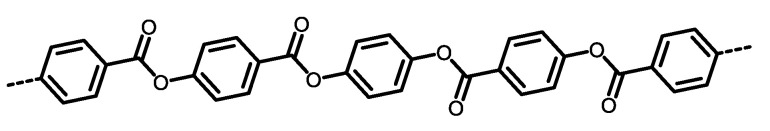
A typical structure incorporated in the PEsIs.

To overcome this difficulty, we proposed modified PIs, poly(ester imide)s (PEsIs) [21,22,23,24,25,26,27], from new monomers [tetracarboxylic dianhydrides (TCDAs) and diamines] with longitudinally extended structures featuring multiple single aromatic units *para*-linked via ester connecting groups, as shown in Scheme 1.

Such PEsIs exhibited low *W*_A_s [21,22,23,24,25,26,27], low CHEs [24,25,26,27], and low dissipation factors (DFs) in the GHz-frequency range [23,24,25,26,27], while maintaining extremely high *T*_g_s and very low CTEs. The *W*_A_ and DF of the resultant PEsI films decreased monotonously with increasing number of aromatic rings in the ester-linked TCDAs (*N*_Ar_). Even when using an ester-linked TCDA with a longitudinally extended structure (*N*_Ar_ = 6), neither gelation during polyaddition nor significant property deterioration, such as a *T*_g_ decrease and a CTE increase, was observed, contrary to our initial concerns. However, a significant decrease in film toughness due to an increase in *N*_Ar_ was unavoidable, even when an ether-containing diamine, effective in improving film toughness, was used [27]. In addition, the flame retardancy tended to deteriorate upon the structural extension [25], probably owing to a concomitant increase in the content of ester connecting groups (*C*_e_) with a relatively low bonding energy. Thus, the PEsIs exhibited certain drawbacks. The improvement in flame retardancy and toughness of conventional aromatic PI films has drawn little attention because the most familiar PMDA/4,4′-ODA-type PI film exhibits both the highest flame retardancy rank (UL-94, V-0 standard [28]) and excellent mechanical strength (toughness) [18], which indicates that these properties require no further improvement.

PEsIs themselves have been studied for a long time. However, in contrast to our PEsI systems, which can be produced via a two-step process consisting of the polyaddition of soluble PEsI precursors followed by solution casting and thermal imidization, classical PEsIs were prepared by esterification via direct polycondensation of imide-containing bisphenols with common dicarbony chlorides [29] or imide-containing dicarbonyl chlorides with common bisphenols [30,31]. Hence, these classical PEsIs themselves needed to be highly soluble in common solvents for subsequent film preparation via solution casting. This solubilization was achieved by introducing highly distorted/non-coplanar units and bulky side groups into the PEsI structures, which prevented the realization of low CTEs and high *T*_g_s. Thus, such classical PEsIs were not considered as dielectric base films for FPCs. Naturally, there was no interest in improving their flame retardancy necessary for FPC applications.

This study describes the structure–property relationships for our various PEsIs from the viewpoint of their FPC applications and elucidates the structural factors for suppressing the deteriorations of their flame retardancy and film toughness.

## 2. Experimental Section

### 2.1. Materials

#### 2.1.1. Monomer Synthesis

**Figure 1:**
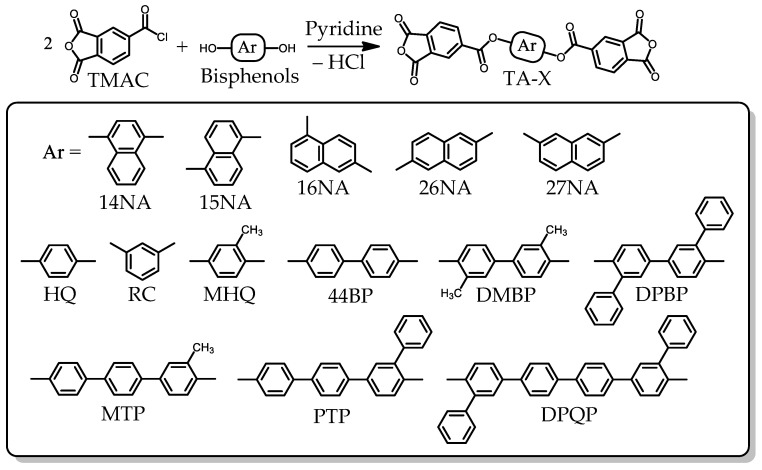
Reaction scheme for synthesis of ester-linked tetracarboxylic dianhydrides (TA-X) using a series of bisphenols.

In this study, numerous TCDAs and diamines were synthesized using the raw materials listed in Table S1. A series of naphthalene-containing TCDAs was synthesized by esterification from trimellitic anhydride chloride (TMAC) and various isomeric dihydroxynathtalenes (DHNAs) in the presence of an HCl acceptor, according to the reaction scheme shown in Figure 1. A typical synthetic procedure is described below. 

**Scheme 2:**
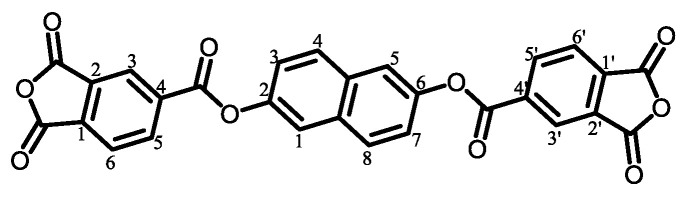
Structure of TA-26NA with numbering.

TA-26NA. In a 200 mL flask, TMAC (4.32 g, 20 mmol) was dissolved in anhydrous tetrahydrofuran (THF, 11.1 mL), and the flask was sealed using a septum rubber stopper. In a separate sealed flask, 2,6-dihydroxynaphthalene (26NA, 1.60 g, 10 mmol) was dissolved in anhydrous THF (4.2 mL) in the presence of pyridine (3.24 mL, 40 mmol). The 26NA solution was gradually added to the TMAC solution kept at 0 °C with a syringe, and the reaction mixture was stirred at 0 °C for several hours, and additionally at room temperature for 24 h. The yellow precipitate formed was collected by filtration and washed with toluene and water to remove the pyridine/HCl salt as a by-product until residual chlorine ion disappeared from the washing using an Ag(NO_3_) aqueous solution (yield: 88%). The crude product containing a partially hydrolyzed portion was heated at 160 °C for 12 h under vacuum to ensure ring closure dehydration. The crude product was purified by recrystallization from anhydrous γ-butyrolactone (GBL). The yellow precipitate (needle crystal) was collected by filtration, washed with toluene, and dried at 160 °C for 12 h under vacuum (recrystallization yield: 79%). The analytical data of this product are as follows. Melting point (mp): a sharp endothermic peak at 302 °C on DSC. FT-IR (KBr plate method, cm^−1^): 3108/3038 (C_Ar_–H stretching vibration), 1858/1793 (acid anhydride group, C=O), 1729 (ester group, C=O). The stretching bands originating from the raw materials used (hydrogen-bonded carboxylic acid O–H at around 2600 cm^−1^ and the phenolic O–H in the 3200–3400 cm^−1^ range) were not observed. ^1^H-NMR [400 MHz, dimethyl sulfoxide (DMSO)-*d*_6_, *δ*, ppm]: 8.71 [dd, 2H (relative integrated intensity: 2.00H), *J* = 7.9, 1.5 Hz, 5,5′-protons of the terminal phthalic anhydride (PAn) groups], 8.68 [d, 2H (2.07H), *J* = 1.4 Hz, 3,3′-protons of PAn], 8.31 [d, 2H (2.08H), *J* = 7.9 Hz, 6,6′-protons of PAn], 8.11 [d, 2H (2.07H), *J* = 8.8 Hz, 4,8-protons of the central 2,6-naphthalene (26NA) unit], 8.05 [d, 2H (2.13H), *J* = 2.5 Hz, 1,5-protons of 26NA], 7.66 [dd, 2H (2.17H), *J* = 8.8, 2.4 Hz, 3,7-protons of 26NA]. Elemental analysis, Anal. Calcd. (%) for C_28_H_12_O_10_ (508.40): C, 66.15; H, 2.38. Found: C, 66.01; H, 2.54. The results confirm that the product is the desired TCDA (TA-26NA) shown in Scheme 2.

Other naphthalene-containing ester-linked TCDAs were synthesized using isomeric DHNAs in a similar manner (Figure 1).

A series of *p*-multi-phenylene-containing TCDAs was synthesized from TMAC and various dihydroxy-*p*-multi-phenylenes. A typical procedure is described below.

**Scheme 3:**
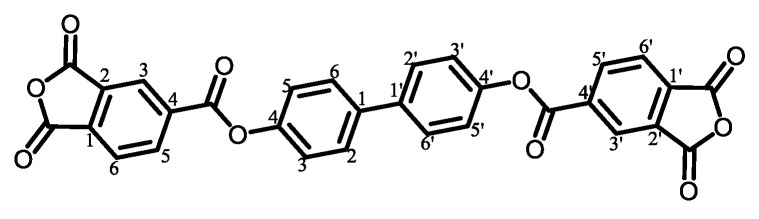
Structure of TA-44BP with numbering.

TA-44BP. In a sealed flask, TMAC (16.85 g, 80 mmol) was dissolved in anhydrous *N*,*N*-dimethylformamide (DMF, 71.3 mL). In a separate sealed flask, 4,4′-biphenol (44BP, 7.45 g, 40 mmol) was dissolved in dry DMF (31.5 mL) in the presence of pyridine (19.4 mL, 240 mmol). The 44BP solution was gradually added to the TMAC solution kept at 0 °C with a syringe, and the reaction mixture was stirred at 0 °C for several hours, additionally at room temperature for 12 h. The yellow precipitate formed was collected by filtration, washed with a small quantity of DMF and a large quantity of water, and dried at 180 °C for 12 h under vacuum (yield: 88%). The crude product was recrystallized from GBL, and the precipitate (golden plate crystal) was washed with GBL and THF, and dried at 200 °C for 12 h under vacuum (recrystallization yield: 84%). The analytical data of this product are as follows. mp: 326 °C (DSC). FT-IR (KBr plate method, cm^−1^): 3104 (C_Ar_–H), 1858/1793 (acid anhydride group, C=O), 1730 (ester group, C=O), 1495 (1,4-phenylene). ^1^H-NMR (400 MHz, DMSO-*d*_6_, *δ*, ppm): 8.68–8.65 [m, 4H (3.98H), 3,3′- and 5,5′-protons of PAn], 8.31 [d, 2H (2.00H), *J* = 7.8 Hz, 6,6′-protons of PAn], 7.85 [d, 4H (4.02H), *J* = 8.6 Hz, 2,2′,6,6′-protons of the central *p*-biphenylene (BP) unit], 7.52 [d, 4H (4.05H), *J* = 8.7 Hz, 3,3′,5,5′-protons of BP]. Elemental analysis, Anal. Calcd. (%) for C_30_H_14_O_10_ (534.44): C, 67.42; H, 2.64. Found: C, 67.66; H, 2.86. The results confirm that the product is the desired TCDA (TA-44BP) shown in Scheme 3.

**Scheme 4:**
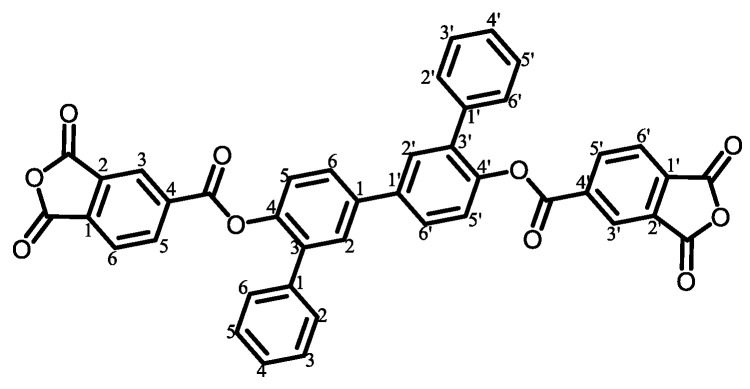
Structure of TA-DPBP with numbering.

TA-DPBP. Another *p*-multi-phenylene-containing ester-linked TCDA (TA-DPBP) was synthesized in anhydrous THF using 4,4′-dihydroxy-3,3′-diphenylbiphenyl (33DP-44BP) instead of 44BP using the same procedures as mentioned above. The crude product was recrystallized from anhydrous acetic anhydride. The analytical data of this product are as follows. mp: 237 °C (DSC). FT-IR (KBr plate method, cm^−1^): 1858/1784 (acid anhydride group, C=O), 1744 (ester group, C=O), 1476 (1,4-phenylene). ^1^H-NMR (400 MHz, DMSO-*d*_6_, *δ*, ppm): 8.53 [dd, 2H (1.86H), *J* = 8.1, 1.1 Hz, 5,5′-protons of PAn], 8.49 [d, 2H (1.62H), *J* = 0.8 Hz, 3,3′-protons of PAn], 8.23 [d, 2H (2.00H), *J* = 8.0 Hz, 6,6′-protons of PAn], 7.96–7.93 [m, 4H (3.94H), 2,2′,6,6′-protons of the central *p*-biphenylene (BP) unit], 7.65–7.63 [m, 6H (6.08H), 2,2′,4,4′,6,6′-protons of the phenyl side groups], 7.41–7.32 [m, 6H (6.32H), 5,5′-protons of BP and 3,3′,5,5′-protons of the phenyl side groups]. The results confirm that the product is the desired TCDA (TA-DPBP) shown in Scheme 4.

Other *p*-multi-phenylene-containing ester-linked TCDAs were synthesized using dihydroxy-*p*-phenylene analogs using the same procedures as mentioned above. (Table S2).

Certain bifunctional flame retardancy-modifiers were synthesized. A typical synthetic procedure is described below. 

**Scheme 5:**
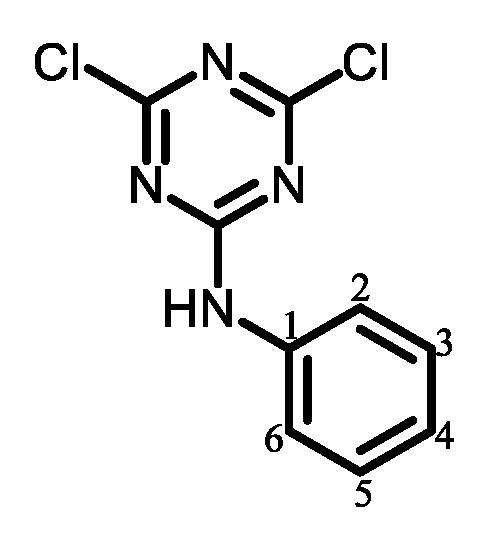
Structure of DC-TAZ with numbering.

PDA-TAZ. This reactive modifier was synthesized with reference to the literature [32] as follows. In a 300 mL flask, cyanuric chloride (CC, 3.79 g, 20.5 mmol) was dissolved in benzene (20 mL). To this solution kept at 0 °C, the mixture of aniline (3.82 g, 41.1 mmol) and benzene (100 mL) in a dropping funnel was gradually added over 4 h; subsequently, the reaction mixture was stirred at room temperature for 12 h. The white precipitate formed (aniline-HCl salt) was removed by filtration. The filtrate (benzene solution) was repeatedly washed with an HCl aqueous solution in a separating funnel to extract/remove an excess of aniline and residual aniline-HCl salt. The benzene solution was dehydrated with MgSO_4_, and the solvent of the filtrate was completely removed using an evaporator. The yielded solid was recrystallized from a mixed solvent of benzene and cyclohexane (6/1, v/v) in a refrigerator, and the precipitate was dried at 60 °C for 12 h under vacuum (yield: 56%). The analytical data of this product are as follows. mp: 133 °C (DSC). FT-IR (KBr plate method, cm^−1^): 3370 (N–H), 3079 (C_Ar_–H), 1605 (C=N), ^1^H-NMR [400 MHz, DMSO-*d*_6_, *δ*, ppm]: 11.16 [s, 1H (1.00H), NH], 7.62 [d, 2H (2.01H), *J* = 7.7 Hz, 2,6-protons of the aniline (AN) unit], 7.41 [t, 2H (2.03H), *J* = 7.6 Hz, 3,5-protons of AN], 7.18 [t, 1H (1.00H), *J* = 7.4 Hz, 4-proton of AN]. The results confirm that the product is the desired intermediate (DC-TAZ) shown in Scheme 5.

**Scheme 6:**
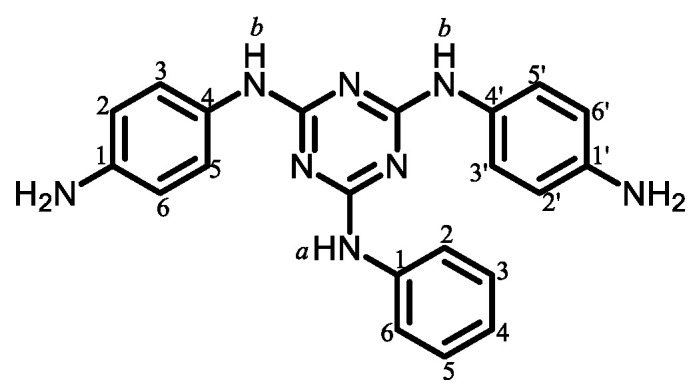
Structure of PDA-TAZ with numbering.

In a three-necked flask, *p*-phenylenediamine (*p*-PDA, 8.75 g, 40.5 mmol) was dissolved in 1,4-dioxane (1,4-DOX, 20 mL) in the presence of Na_2_CO_3_ (0.53 g, 5 mmol). To this solution kept at 100 °C, a 1,4-DOX solution (100 mL) of DC-TAZ (1.15 g, 5.01 mmol) in a dropping funnel was gradually added over 2 h, and the reaction mixture was refluxed at 100 °C for 4 h in an N_2_ atmosphere. An undissolved residue (Na_2_CO_3_) was removed by filtration. The filtrate was gradually poured into a large quantity of water to remove an excess of *p*-PDA, and the pale-red precipitate formed was repeatedly washed with water and hot toluene, and dried at 120 °C for 3 h under vacuum (yield: 72%). The analytical data of this product are as follows. mp: 222 °C (DSC). FT-IR (KBr plate method, cm^−1^): 3389/3317/3249 (NH_2_ + N–H), 3023 (C_Ar_–H), 1619 (C=N). ^1^H-NMR [400 MHz, DMSO-*d*_6_, *δ*, ppm]: 8.94 [s, 1H (1.00H), NH*_a_*], 8.63 [s, 2H (2.01H), NH_b_], 7.79 [d, 2H (2.05H), *J* = 5.2 Hz, 2,6-protons of AN], 7.32 [d, 4H (4.00H), *J* = 7.6 Hz, 3,3′,5,5′-protons of the *p*-PDA unit], 7.22 [t, 2H (2.01H), *J* = 7.6 Hz, 3,5-protons of AN], 6.92 [t, 1H (1.08H), *J* = 7.3 Hz, 4-proton of AN], 6.51 [d, 4H (4.11H), *J* = 8.6 Hz, 2,2′,6,6′-protons of the *p*-PDA units], 4.78 [s, 4H (4.12H), NH_2_]. The results confirm that the product is the desired compound (PDA-TAZ) shown in Scheme 6.

**Scheme 7:**
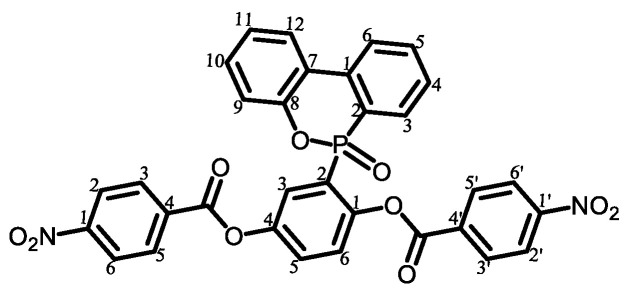
Structure of NB-HCAHQ with numbering.

AB-HCAHQ. In a sealed flask, 4-nitrobenzoyl chloride (4-NBC, 8.35 g, 45 mmol) was dissolved in anhydrous DMF. In a separate sealed flask, HCA-HQ (6.49 g, 20 mmol) was dissolved in anhydrous hot DMF (16 mL) in the presence of pyridine (7.4 mL, 120 mmol). To the 4-NBC solution, the HCA-HQ solution was added at room temperature, and the reaction mixture was stirred at room temperature for 12 h. The white precipitate was collected by filtration, washed using a small quantity of DMF and water, and dried at 110 °C for 12 h under vacuum. The analytical data of this product is as follows. mp: 272 °C (DSC). FT-IR (KBr plate method, cm^−1^): 3111 (C_Ar_–H), 1748 (ester group, C=O), 1528/1348 (NO_2_), 1242 (P=O) [33], 1181 (P–O–Ar) [33]. ^1^H-NMR [400 MHz, DMSO-*d*_6_, *δ*, ppm]: 8.47–8.42 [m, 5H (4.38H), 2,2′,6,6′-protons of the terminal nitrobenzene (NB) units + 6-proton of the pendant phosphaphenanthrene (PPN) unit], 8.16 [dd, 1H (0.89H), *J* = 14.6, 2.8 Hz, 12-proton of PPN], 8.05 [d, 2H (1.95H), *J* = 8.8 Hz, 3′,5′-protons of NB], 8.01–7.98 [m, 1H (0.91H), 3-proton of PPN], 7.91–7.88 [m, 2H (1.92H), 3,5-protons of NB], 7.73 [t, 1H (0.88H), *J* = 7.2 Hz, 4-proton of PPN], 7.67–7.64 [m, 1H (0.81H), 5-proton of PPN], 7.61–7.52 [m, 4H (3.80H), 3- + 5- + 6-protons of the central hydroquinone (HQ) unit], 7.40 [t, 1H (1.00H), *J* = 7.6 Hz, 10-proton of PPN], 7.23 [d, 1H (0.98H), *J* = 8.2 Hz, 9-proton of PPN], 7.14 [t, 1H (0.96H), *J* = 7.6 Hz, 11-proton of PPN]. The results suggest that the product is the desired intermediate (NB-HCAHQ) shown in Scheme 7. 

**Scheme 8:**
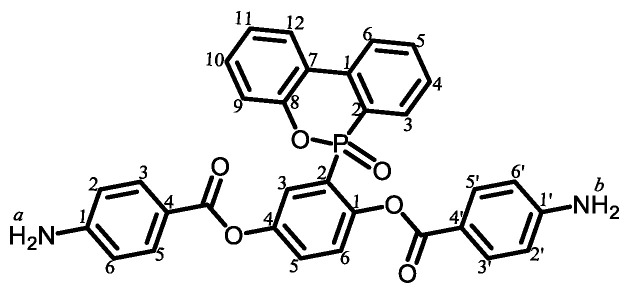
Structure of AB-HCAHQ with numbering.

In a three-necked flask, NB-HCAHQ (3.74 g, 6 mmol) was dissolved in anhydrous DMF (35 mL) in the presence of Pd/C (0.374 g) as a catalyst. The catalytic reduction at 100 °C in a hydrogen atmosphere was unsuccessful because of an unknown decomposition. Therefore, the reaction was conducted at room temperature for 12 h while monitoring the progress of the reaction by thin layer chromatography. The residual catalyst was removed by filtration, and the filtrate was gradually poured into a large quantity of water. The white precipitate yielded was collected by filtration, washed with hot 1,4-DOX, and dried at 120 °C for 12 h under vacuum (yield: 90%). The crude product was recrystallized twice from DMF and dried at 120 °C for 12 h under vacuum (recrystallization yield: 67%). The analytical data of this product are as follows. mp: 296 °C (DSC). FT-IR (KBr plate method, cm^−1^): 3451/3358/3241 (NH_2_ group, N–H), 1709 (ester group, C=O), 1516 (1,4-phenylene group), 1246 (P=O) [33], 1165 (P–O–Ar) [33]. ^1^H-NMR [400 MHz, DMSO-*d*_6_, *δ*, ppm]: 8.07–8.04 [m, 2H (1.97H), 3′,5′-protons of the terminal aniline (AN) unit], 7.88–7.83 [m, 3H (2.92H), 3,5-protons of AN + 6-proton of PPN], 7.71–7.64 [m, 2H (1.92H), 12- + 3-protons of PPN], 7.53–7.37 [m, 4H (3.85H), 5-proton of PPN and 3- + 5- + 6-protons of HQ], 7.26–7.24 [m, 2H (1.97H), 4- + 10-protons of PPN], 6.99–6.97 [m, 2H (1.98H), 9- + 11-protons of PPN], 6.66 [d, 2H (2.00H), *J* = 8.8 Hz, 2′,6′-protons of AN], 6.26–6.21 [m, 4H (3.90H), 2,6-protons of AN + NH_2_ (*b*)], 6.07 [s, 2H (1.83H), NH_2_ (*a*)]. Elemental analysis, Anal. Calcd. (%) for C_32_H_23_O_6_N_2_P (562.52): C, 68.33; H, 4.12; N, 4.98. Found: C, 68.20; H, 4.30; N, 4.40. The results correspond to the structure of the desired diamine (AB-HCAHQ) shown in Scheme 8.

**Scheme 9:**
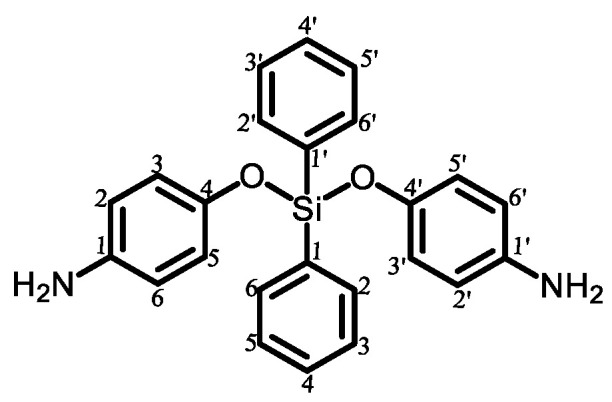
Structure of AP-DPSi with numbering.

AP-DPSi. In a sealed three-necked flask, 4-aminophenol (4-AP, 2.40 g, 22 mmol) was dissolved in anhydrous THF (50 mL) in the presence of triethylamine (TEA, 3.36 mL, 24.2 mmol). To this solution, dichlorodiphenylsilane (DCDPSi, 2.07 mL, 10 mmol) dissolved in THF (80 mL) was gradually added with a syringe, and the reaction mixture was refluxed at 75 °C for 3 h in an N_2_ atmosphere. The precipitate yielded (TEA–HCl salt as a by-product) was removed by filtration, and the filtrate was concentrated using an evaporator. Diethyl ether was then added to the precipitate to extract the desired product, and an undissolved portion (residual 4-AP) was removed by filtration. The ether solution was concentrated and recrystallized in a freezer. The precipitate formed was dried at 80 °C for 10 h under vacuum (yield: 27%). The analytical data of this product are as follows. mp: 103 °C (DSC). FT-IR (KBr plate method, cm^−1^): 3403/3328/3216 (NH_2_ group, N–H), 1125 (O–Si–O). ^1^H-NMR [400 MHz, DMSO-*d*_6_, *δ*, ppm]: 7.65 [d, 4H (4.14H), *J* = 6.8 Hz, 2,2′,6,6′-protons of the Si-Phenyl unit], 7.49–7.38 [m, 6H (6.24H), 4,4′- and 3,3′,5,5′-protons of the Si-phenyl unit], 6.67 [d, 4H (4.00H), *J* = 8.8 Hz, 3,3′,5,5′-protons of the aniline (AN) unit], 6.39 [d, 4H (4.18H), *J* = 8.6 Hz, 2,2′,6,6′-protons of AN], 4.68 [s, 4H (3.95H), NH_2_]. The results confirm that the product is the desired diamine (AP-DPSi) shown in Scheme 9.

**Scheme 10:**
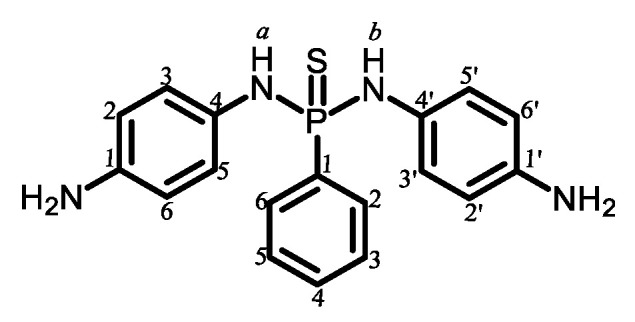
Structure of PDA-PTP with numbering.

PDA-PTP. In a three-necked flask, *p*-PDA (8.75 g, 40 mmol) was dissolved in anhydrous hot 1,4-DOX (40 mL) in the presence of TEA (4.17 mL). To this solution, phenylthiophosphonic dichloride (PTP-DC, 0.703 g, 5 mmol) dissolved in 1,4-DOX (80 mL) was gradually added over 4 h using a dropping funnel, and the reaction mixture was refluxed at 100 °C for 4 h in an N_2_ atmosphere. After the precipitate yielded (TEA-HCl salt) was filtered off, the filtrate was concentrated using an evaporator. The black precipitate formed was repeatedly washed with water to remove an excess of *p*-PDA and dried at 100 °C for 12 h under vacuum (yield: 75%). The analytical data of this product are as follows. mp: a broad peak at 250 °C (DSC). FT-IR (KBr plate method, cm^−1^): 3335/3210 (NH_2_ group, N–H stretching vibration), 1620 (NH_2_ deformation), 1512 (1,4-phenylene unit), 828 (P=S) [33]. ^1^H-NMR [400 MHz, DMSO-*d*_6_, *δ*, ppm]: 7.87 [d, 1H, *J* = 6.4 Hz, 2 (6)-proton of the P-linked phenyl unit], 7.84 [d, 1H, *J* = 6.4 Hz, 6 (2)-proton of the P-linked phenyl unit], 7.50–7.45 [m, 3H (3.38H), 3,5-protons + 4-proton of the P-linked phenyl unit], 7.17 [s, 1H (0.95H), NH*_a_*], 7.15 [s, 1H (0.95H), NH*_b_*], 6.84 [d, 4H (3.98H), *J* = 8.6 Hz, 3,3′,5,5′-protons of AN], 6.37 [d, 4H (4.05H), *J* = 8.6 Hz, 2,2′,6,6′-protons of AN], 4.62 [s, 4H (4.00H), NH_2_]. The results confirm that the product is the desired diamine (PDA-PTP) shown in Scheme 10.

#### 2.1.2. Common Monomers

**Figure 2:**
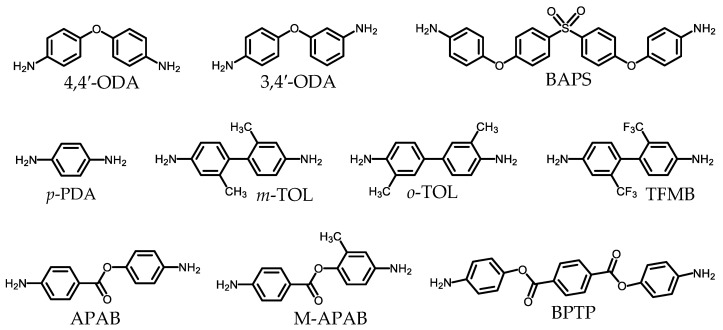
Structures and abbreviations of the common diamines used in this study.

The commercial sources, melting points, and abbreviations of the common monomers used (Figure 2) are listed in Table S3.

#### 2.1.3. Polyaddition and Thermal Imidization for PEsI Film Preparation

**Figure 3:**
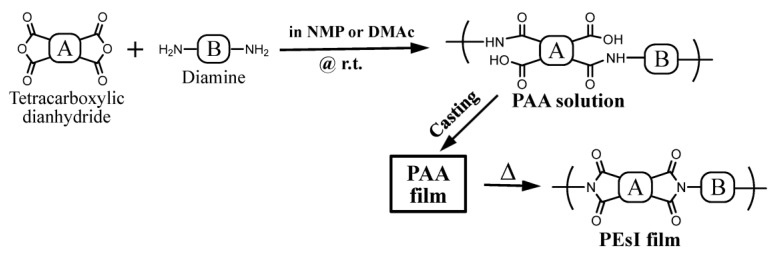
Schemes of polyaddition, solution casting of PAAs, and thermal imidization for PEsI film preparation.

The PEsI films were prepared via the conventional two-step process, including the equimolar polyaddition of TCDAs and diamines at room temperature, solution casting of the PEsI precursors [poly(amic acid)s (PAAs)], and thermal imidization of the PAA films, according to the scheme shown in Figure 3.

Polyaddition was conducted as follows. In a sealed vial, TCDA solid (2 mmol) was added in one portion to an *N*-methyl-2-pyrrolidone (NMP) solution of a diamine (2 mmol) with continuous magnetic stirring, and the reaction mixture (initial solid content: 25 or 30 wt%) was stirred at room temperature until it became homogeneous and had a maximum solution viscosity (typically after 72 h). The reaction mixture was, as appropriate, gradually diluted with a minimal quantity of the same solvent to ensure effective magnetic stirring.

**Figure 4:**
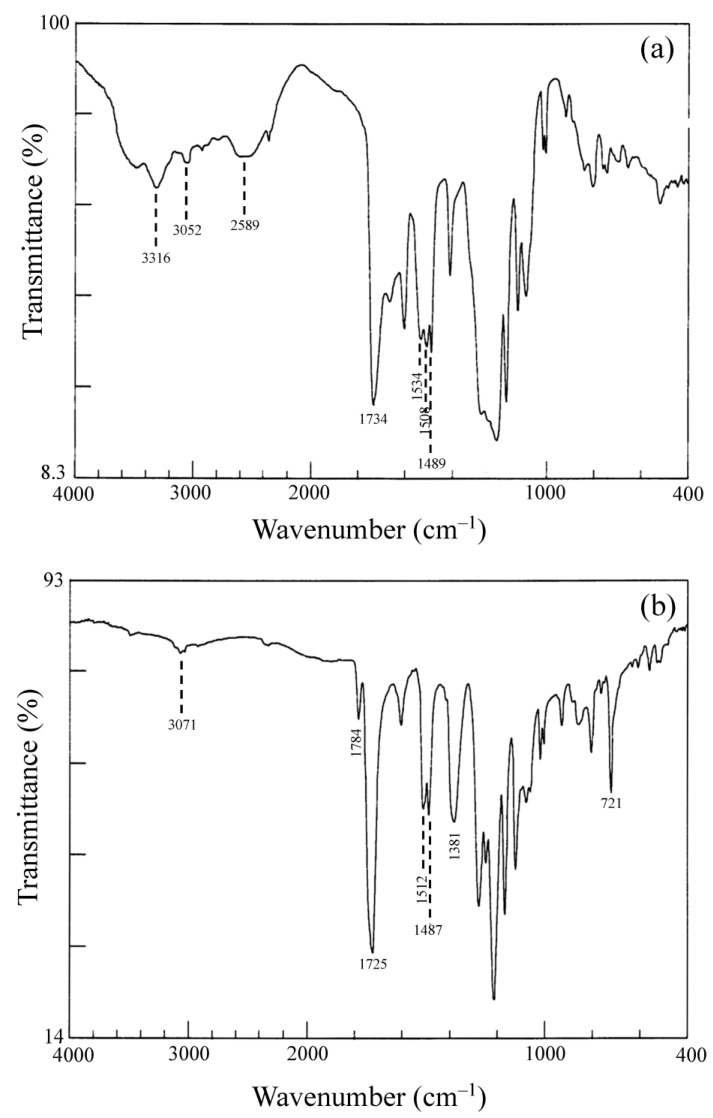
FT-IR spectra of thin films for TA-MTP/APAB(85);4,4′-ODA(15): (**a**) PAA and (**b**) PEsI.

The transmission FT-IR spectra confirmed the formation of PAAs using separately prepared thin cast films (4–5 μm thick) with a non-uniform thickness to erase the interference fringes. A typical spectrum is shown in Figure 4a. The spectrum includes the following IR bands (cm^−1^): 3316 (amide groups, N–H stretching vibration band), 3052 (C_Ar_–H), 2589 (hydrogen-bonded COOH groups, O–H), 1734 (ester groups, C=O), 1534 (amide groups, C=O), 1508/1489 (1,4-phenylene units). Additionally, an unnecessary IR band at 1860 cm^−1^ due to the acid anhydride groups was not observed.

The PEsI films (typically 25 μm thick) were prepared via thermal imidization of the PAA cast films as follows. The PAA solutions obtained via polyaddition were bar-coated on a glass substrate and dried at 80 °C for 3 h in an air-convection oven. Thermal imidization was conducted by stepwise heating the PAA cast films on the substrate at established imidization temperatures (*T*_i_), i.e., 250 °C for 1 h + 300 °C for 1 h under vacuum, and finally annealed at *T*_ann_ = 350 °C for 1 h under vacuum without the substrate to remove residual stress. In some cases, these thermal conditions were adjusted to obtain better-quality flexible films without residual strain.

The transmission FT-IR spectra confirmed the completion of thermal imidization using the above-mentioned thin films. A typical spectrum is shown in Figure 4b. The spectrum includes the following IR bands (cm^−1^): 3071 (C_Ar_–H), 1784 (imide groups, C=O), 1725 (imide and ester groups, C=O), 1512/1487 (1,4-phenylene units), 1381 (imide groups, N–C_Ar_), and 721 (imide groups, ring deformation). Additionally, the unnecessary IR bands characteristic of PAAs (e.g., the amide C=O stretching band at ~1660/1530 cm^−1^ and the hydrogen-bonded carboxylic acid O–H stretching band at ~2600 cm^−1^) completely disappeared after the thermal imidization.

In this study, the PAA and the corresponding PEsI systems are abbreviated using the symbols of the TCDAs (A) and diamines (B) as A/B for homopolymers and A1;A2/B1;B2 for copolymers.

### 2.2. Measurements and Characterization

#### 2.2.1. Structural Characterization of Ester-Linked TCDAs 

The structures of TCDAs and flame retardancy-modifiers synthesized in this study and their intermediates were characterized by FT-IR (JASCO, Tokyo, Japan, FT/IR 5300 or FT/IR 4100 infrared spectrometer), ^1^H-NMR spectroscopy (JEOL, Tokyo, Japan, ECP400), and elemental analysis (J-Science Lab, Kyoto, Japan, Micro Corder JM10). Their melting points were determined from the endothermic peak temperatures by differential scanning calorimetry (Netzsch Japan, Yokohama, Japan, DSC3100) or differential temperature analysis (Netzsch Japan, Yokohama, Japan, TG-DTA2000 or TG-DTA2000S) with a heating rate of 5 °C min^−1^ in an N_2_ atmosphere.

#### 2.2.2. Reduced Viscosities

The inherent viscosities (*η*_inh_) of PAAs are difficult to determine by extrapolation to infinite dilution, owing to the polyelectrolyte effect. Instead, the reduced viscosities (*η*_red_) of PAAs were determined at a solid content of 0.5 wt% at 30 °C on an Ostwald viscometer by prompt dilution of the as-polymerized PAA solutions with the same solvents.

#### 2.2.3. Linear Coefficients of Thermal Expansion

The linear coefficient of thermal expansion (CTE) is an index representing the *XY*-direction thermal dimensional stability of film samples in the glassy temperature range (*T* < *T*_g_). Thermal mechanical analysis (TMA) was conducted to measure the CTEs of the PEsI films on a thermomechanical analyzer (Netzsch Japan, Yokohama, Japan, TMA4000). The CTEs were determined as an average in the range of 100–200 °C with a fixed load (0.5 g per unit film thickness in μm, e.g., 12.5 g load for a 25 μm-thick film) using a fixed size of specimens (length: 20 mm, width: 5 mm, typical thickness: 25 μm, and chuck-to-chuck length: 15 mm). Before the measurements, the specimens were once heated to 150 °C in the TMA chamber while flowing dry N_2_ to remove the adsorbed water, and cooled to room temperature. After the specimen length was reset to 0% elongation at room temperature, the second heating run was conducted with a heating rate of 5 °C min^−1^ under a dry N_2_ flow (500 mL min^−1^) for data collection.

#### 2.2.4. Linear Coefficients of Hygroscopic (Humidity) Expansion

The linear coefficient of hygroscopic (humidity) expansion (CHE) is an index representing the *XY*-direction dimensional stability at room temperature against water/moisture absorption. The CHEs of the PEsI films were determined from the dimensional changes of the specimens during storage at a relative humidity (RH) of 80% using the above-mentioned TMA instrument connected to a precision humidity generator (Shinyei Technology, Kobe, Japan, SRG-1R-1). To avoid dew formation in the chamber, the relative humidity of the introduced wet N_2_ gas was gradually increased from 50 to 70%RH in a 10%RH interval (held for 5 min at each step), finally being maintained at 80%RH. The elongation–time curves at 80%RH were monitored until the elongation values leveled off (typically after 15–18 h).

#### 2.2.5. Glass Transition Temperatures 

Dynamic mechanical analysis (DMA) was conducted in a dry N_2_ atmosphere at a heating rate of 5 °C min^−1^ and a sinusoidal load frequency of 0.1 Hz with an amplitude of 15 gf to measure the storage modulus (*E*′) and loss energy (*E*″) of the PEsI films as a function of temperature using the above-mentioned TMA instrument. The glass transition temperatures (*T*_g_s) (or *α*-transition temperatures, *T*_α_) of the PEsI films were determined from the main peak temperatures in the *E*″ curve, unless otherwise noted (method-1). In some cases, if any, the sub-*T*_g_s (or *β*-transition temperatures, *T*_β_) of the PEsI films were determined from the secondary peak temperatures in the *E*″ curve.

The *T*_g_s were also determined from the inflection point of the TMA curve measured under a fixed static load, i.e., the point where the specimens started to elongate abruptly, as the intersection of two tangential lines (method-2). The TMA-based *T*_g_s were essentially equivalent to the DMA-based ones, although the former tended to be slightly higher than the latter.

#### 2.2.6. Thermal Decomposition Temperatures

The 5% weight loss temperatures (*T*_d_^5^) of the PEsI films, which are the indexes representing thermal and thermo-oxidative stability, were determined by thermogravimetric analysis (TGA) at a heating rate of 10 °C min^−1^ in a dry N_2_ and/or air atmosphere on a thermo-balance (Netzsch Japan, Yokohama, Japan, TG-DTA2000 or TG-DTA2000S). A small weight loss based on water desorption at ~100 °C during the heating runs was compensated by an offset at 150 °C to 0% weight loss for the data analysis.

#### 2.2.7. Mechanical Properties

The tensile properties, including modulus (*E*), strength (*σ*_b_), and elongation at break (*ε*_b_), of the PEsI films (specimen length: 30 mm, width: 3 mm, thickness: typically 25 μm, chuck-to-chuck length: 20 mm, valid sample number: *n* = 10–20) were evaluated on a mechanical testing machine (A & D, Tokyo, Japan, Tensilon UTM-II) at a cross-head speed of 8 mm min^−1^ at room temperature. The raw data were analyzed using a data processing program (Softbrain, Tokyo, Japan, UtpsAcS Ver. 4.09).

#### 2.2.8. Water Uptake

The water uptake (*W*_A_) of the PEsI films was estimated according to the JIS K 7209 standard [34] using Equation (1):

*W*_A_ = [(*W* − *W*_0_)/*W*_0_] × 100 (1)

where *W*_0_ is the weight of a film specimen vacuum-dried at 50 °C for 24 h, and *W* is the weight of the specimen immersed in water at 23 °C for 24 h and carefully blotted dry with tissue paper. A 50 μm thick KAPTON^®^ H film was used as a reference sample (*W*_A_ = 2.7% [18]) in each measurement of the specimens.

#### 2.2.9. Contents of Imide, Methyl, Ester, and Phenylene Ether Groups in the Main Chains

The contents of imide group (O=C–N–C=O) in the PEsI (*C*_i_, wt%) were calculated using Equation (2):

*C*_i_ = [*F*_W_ (imide)/*F*_W_ (unit)] × 100 (2)

where *F*_W_ (imide) and *F*_W_ (unit) denote the formula weights of the imide group and repeating units, respectively. 

The contents of methyl (*C*_m_), ester (*C*_e_), and phenylene ether groups (*C*_PhO_) were calculated in a similar manner using the following equations:

*C*_m_ = [*F*_W_ (CH_3_)/*F*_W_ (unit)] × 100 (3)

*C*_e_ = [*F*_W_ (COO)/*F*_W_ (unit)] × 100 (4)

*C*_PhO_ = [*F*_W_ (PhO)/*F*_W_ (unit)] × 100 (5)

where *F*_W_ (CH_3_), *F*_W_ (COO), and *F*_W_ (PhO) represent the formula weights of methyl, ester, and phenylene ether groups, respectively.

#### 2.2.10. Thickness-Direction Birefringence

To estimate the relative degree of in-plane chain orientation, the thickness-direction birefringence (Δ*n*_th_) of the PEsI films was measured on an Abbe refractometer (Atago, Tokyo, Japan, 4T, *n*_D_ range: 1.47–1.87) equipped with a sodium lamp at 589.3 nm (*D*-line) and a polarizer using a contact liquid (sulfur-saturated methylene iodide *n*_D_ = 1.78–1.80) and a test piece (*n*_D_ = 1.92). The Δ*n*_th_ was calculated from the following relationship:

Δ*n*_th_ = *n*_xy_ − *n*_z_ (6)

where *n*_xy_ and *n*_z_ denote the refractive indices at 589.3 nm in the *XY*- and *Z*-directions, respectively. 

#### 2.2.11. Flame Retardancy

The flame retardancy of the PEsI films was evaluated according to the UL-94 vertical burning test [28] using a set of five specimens [length: 125 mm, width: 13 mm, and average thickness (*d*): ~20–30 μm], as shown in Figure S1a. The test was conducted by bringing a 20 mm flame into contact with the lower end of the specimen for 10 s twice. The samples were certified as having passed the V-0 standard [28] when none of the specimens burned out up to the clamped top edge within a combustion time of 10 s and no flaming melt-down fragments were observed in any case, as typically shown in Figure S1b. The ratings of the UL-94V tests (V-0, V-1, and V-2) are explained in Table S4.

Although the limiting oxygen index (LOI) [35,36,37,38] is a useful indicator of flame retardancy, we did not conduct LOI measurements because of the lack of the required apparatus.

#### 2.2.12. Morphological Observation of Carbonized Portion after Burning Test

Morphology of the carbonized portion after the burning tests was observed on a scanning electron microscope (SEM, ELIONIX Inc., Tokyo, Japan, EXM-3500). The samples were precoated with gold by vacuum deposition using a quick spattering coater (ELIONIX Inc., SC-701).

## 3. Results and Discussion

### 3.1. Difficulty in Simultaneously Achieving Several Target Properties

As mentioned above, new heat-resistant dielectric materials exhibiting several beneficial properties are in high demand. Table 1 qualitatively represents the difficulty in achieving certain property combinations. For example, the simultaneous realization of high *T*_g_ and high thermal decomposition temperature (*T*_d_) is relatively easy [blue solid circle (●) in Table 1] because these properties require similar molecular designs; these properties tend to be enhanced with increasing contents of rigid aromatic and benzimide units. A similar situation is observed for other combinations, such as high *T*_g_–high flame retardancy (●) and high *T*_g_–low CTE property (●). In contrast, the simultaneous realization of high *T*_g_ and high *ε*_b_ is difficult (♦) because these properties require mutually opposite molecular design directions; the latter is enhanced by flexible main-chain structures necessary for chain entanglement, whereas the former is not. The simultaneous achievement of low CTE and high *ε*_b_ is most difficult (⁕) because the former requires rigid/linear main-chain structures, contrary to the latter. Due to a similar reason, it is extremely challenging to simultaneously achieve low CTE and high thermoplasticity (⁕) [39]. High optical transparency cannot be easily combined with high flame retardancy, as suggested by the fact that most of colorless resins have poor flame retardancy, owing to their high contents of flammable aliphatic units [40,41,42].

In PI systems, a similar trade-off relationship is observed between optical transparency and flame retardancy. Except for a wholly aromatic PI derived from 4,4′-(hexafluoroisopropylidene)diphthalic anhydride (6FDA) and 2,2′-bis(trifluoromethyl)benzidine (TFMB) [43], colorless PI films are produced using at least one aliphatic monomer (TCDA, diamine, or both) [44]. Such semi-cycloaliphatic PIs usually have poor flame retardancy [45,46].

Thus, from the difficulty of concurrently achieving even two desired properties in some cases, it is clear that the simultaneous realization of multiple target properties, as described in the present study, is a formidable challenge.

### 3.2. Difficulty in Enhancing the Flame Retardancy of PEsI Systems

PEsIs obtained using TCDAs with longitudinally extended structures, in which multiple aromatic units were *para*-linked via ester connecting groups, are limited systems that exhibit significantly reduced *W*_A_s, CHEs, and DFs at 10 GHz while maintaining very high *T*_g_s and low CTEs [23,24,25,26,27]. However, excessive structural extension via ester groups resulted in discernible decreases in film toughness [27] and chemical heat resistance [decreases in *T*_d_^5^ (N_2_) and *T*_d_^5^ (air)] [24,27]. The decreased *T*_d_^5^s are probably due to the lower bonding energy of the ester group than that of the commonly used aromatic ether (–Ph–O–Ph–) group [47]. A deterioration in flame retardancy, in relation to the *T*_d_^5^ reduction, was also observed [24]. 

Table 2 presents a typical example of the prominent influence of the ester connecting groups on flame retardancy; unlike a conventional wholly aromatic home-made PI film (#1) obtained from 3,3′,4,4′-biphenyltetracarboxylic dianhydride (*s*-BPDA) and 4,4′-ODA, a related PEsI film (#2), which corresponds to its modified version by copolymerization with an ester-containing diamine (AB-HQ), did not meet V-0 [25]. The decreased flame retardancy of the latter was probably ascribed to the incorporated ester groups and the resulting decrease in the content of benzimide units. This finding suggests that serious flame retardancy deterioration may be unavoidable for numerous PEsIs.

### 3.3. Strategies for Mitigating Flame Retardancy Deterioration

A variety of flame retardants are available, and their effects and the mode of action have been proposed [40,41,48,49]. First, we reviewed the classification and effects of typical flame retardants often used in common polymer systems (Figure S2). Combustion of polymers requires the combination of flammable materials as the fuels, oxygen supply, and heat transfer. Therefore, the flame retardancy of polymeric materials can be dramatically enhanced by hindering at least one of these factors [49]. 

The low-cost and effective inorganic flame retardants are usually dispersed into thermoplastic resins by melt-kneading. For example, metal hydroxides such as Mg(OH)_2_ and Al(OH)_3_ impart flame retardancy by undergoing their endothermic dehydration (cooling effect) at elevated temperatures during combustion and diluting the generated combustible gases with the by-produced water vapor. However, to achieve a meaningful effect, very high contents of these inorganic flame retardants need to be present, which results in a significant *W*_A_ increase and a deterioration in electrical insulation reliability. Therefore, such inorganic flame retardants are not suitable for PEsIs used in FPC applications.

In contrast, halogenated (typically brominated) organic flame retardants [and their combinations with flame retardancy promoters (typically Sb_2_O_3_)] and (in)organic phosphorus flame retardants are effective even at low contents. It is believed that the phosphorus flame retardants have an oxygen-blocking effect of dense poly(phosphoric acid) and poly(metaphosphoric acid) layers formed during the thermal decomposition of phosphorus flame retardants, an oxygen-/heat-blocking effect of the subsequently formed char layers, and an effect on trapping the highly reactive OH and H radicals generated in the gas phase. However, these additive-type flame retardants are not suitable for our PEsI systems because they are prone to phase separation and migration, resulting in undesirable bleed-out. Although reactive halogenic (typically brominated) flame retardants suppress the bleed-out, some of them are subject to the RoHS regulation [40]. On the other hand, reactive phosphorus-containing organic flame retardants [24,46,50,51,52] can be applied to our PEsI systems, if used at small loadings as comonomers.

### 3.4. Approaches to Improve the Flame Retardancy of PEsIs Using Reactive Modifiers

In this study, we initially investigated the promotional effects of various bifunctional modifiers on the flame retardancy of a selected PEsI system, TA-MHQ/BPTP(60);4,4′-ODA(40) copolymer. The properties of the pristine and the modified systems are summarized in Table 3. 

The pristine PEsI (#3) exhibited excellent combined properties, i.e., an extremely high *T*_g_ (390 °C), very low CTE (12.3 ppm K^−1^), low *W*_A_ (0.52%), and very low CHE (5.0 ppm/%RH) while maintaining good film toughness (*ε*_b max_ = 41.6%). The observed low CTE, corresponding to a high Δ*n*_th_ of this film (0.137), is ascribed to a high extent of chain alignment in the film-plane (*XY*) direction (in-plane chain orientation), which is induced upon the thermal imidization of the PAA cast films fixed on the substrates [53,54,55,56,57,58,59,60,61,62]. However, this PEsI copolymer had insufficient flame retardancy (incompatible with the V-0 standard), as indicated by the fact that, in the vertical burning tests, the specimens very rapidly burned out up to the clamped top edge with a large flame. As discussed later, the results involve the presence of methyl side groups and the high content of ester groups.

We initially selected a naphthalene-containing TCDA [1,4,5,8-naphthalenetetracarboxylic dianhydride (1,4,5,8-NTDA)] as the reactive modifier. This idea was inspired by the fact that high-temperature treatment of polyperinaphthalene, which was prepared from 1,4,5,8-NTDA as the starting material, produced graphite [63,64]. The incorporation of a small quantity (5 mol%) of 1,4,5,8-NTDA-based diimide units into the main chains was expected to facilitate the formation of oxygen/heat-blocking char layers. This modification (#4*x*) resulted in a discernibly decreased *η*_red_, compared with that of the pristine PEsI system (#3). Copolymerization with 10 mol% of 1,4,5,8-NTDA (#4*y*) further lowered the *η*_red_, as shown in Table 3. The results are ascribed to the inherently low reactivity of 1,4,5,8-NTDA [65], originating from its stable six-membered acid anhydride ring. The discernibly increased CTE at 10 mol% of 1,4,5,8-NTDA is probably related to the significantly decreased *η*_red_ (molecular weight) of the corresponding PAAs [23]. The somewhat decreased film toughness also results from the decreased molecular weight, which causes a decrease in the extent of chain entanglement [66]. In addition, *W*_A_ gradually increased with increasing 1,4,5,8-NTDA content, probably owing to a concomitant increase in imide group content and a decrease in ester group content. Even at 10 mol% of 1,4,5,8-NTDA (#4*y*), no clear improvement in flame retardancy was observed.

In the system using 2,3,6,7-NTDA (#5*x*, 5*y*), the *T*_g_ and CTE remained virtually unchanged. On the other hand, a slight toughening effect was observed, probably owing to the increase in *η*_red_ (molecular weight) due to the high polyaddition reactivity of 2,3,6,7-NTDA based on its five-membered functional groups [65]. However, the 2,3,6,7-NTDA-modified systems (5 and 10 mol%), as well as their 1,4,5,8-NTDA-modified counterparts, were ineffective in improving flame retardancy. We did not attempt to further increase the modifier content because serious deteriorations of the properties (*W*_A_, CHE, etc.) are predicted.

We also used a siloxane-containing modifier, AP-DPSi [5 mol% (#6*x*) and 10 mol% (#6*y*)], expecting a flame-resistant effect similar to that of siloxane polymers [42,48,49]. However, this approach was ineffective and did not significantly alter other properties.

Triazine compounds (melamine derivatives) often impart flame retardancy [42,48,49]. The modification with 10 mol% PDA-TAZ (#7*y*) slightly increased the CTE, *W*_A_, and CHE and decreased the *ε*_b_. Even though the PDA-TAZ-modified films (#7*x*, 7*y*) remained V-0-incompatible, a certain effect was observed; they exhibited milder (slower) combustion, unlike the violent (rapid) burning-out with a large flame observed for the pristine system (#3). 

Phosphorus compounds efficiently promote flame retardancy [24,40,41,42,45,46,48,49,50,51,52]. The introduction of PDA-PTP (#8*x*, 8*y*) with a P=S group slightly increased the CTE and *W*_A_. The observed *W*_A_ increase is very likely attributed to the polar NH groups in this modifier. A positive effect was also observed; the *T*_d_^5^ (N_2_) and *T*_d_^5^ (air) discernibly increased. This behavior was observed only when using PDA-PTP among the modifiers examined, suggesting its specific effect on suppressing thermal decomposition at an early stage. Nonetheless, the PDA-PTP-modified system remained V-0-incompatible, although the specimens showed milder combustion than those of the pristine system (#3).

Table 3 lists the detailed properties of AB-HCAHQ-modified PEsIs with different modifier contents. In contrast to the PDA-PTP-modified system (#8), the use of AB-HCAHQ (#9) enhanced neither *T*_d_^5^ (N_2_) nor *T*_d_^5^ (air). Nonetheless, copolymerization with only 5 mol% of AB-HCAHQ (#9*x*), which corresponds to a very low phosphorus content (*C*_P_) of 0.21 wt% in the resin, dramatically improved flame retardancy (V-0 passed) without significantly sacrificing the excellent combined properties of the original PEsI (#3). The results also correspond to a discernibly increased residual weight at 650 °C (RW_650_ = 28.7% in air) during TGA for #9*x*, compared with that of the pristine PEsI (#3) (RW_650_ = 13.5%). 

**Figure 5:**
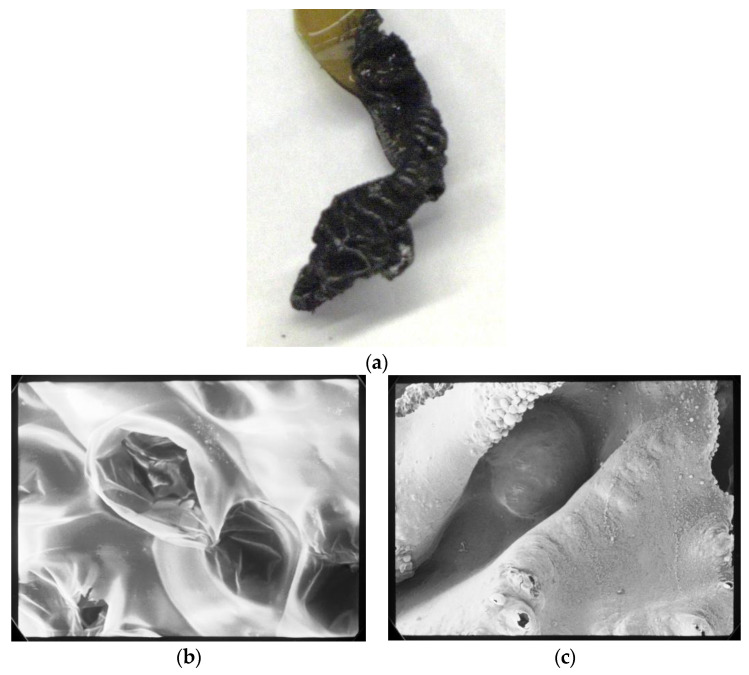
Appearance of the specimens after the UL-94 vertical burning test for TA-MHQ/BPTP(50);4,4′-ODA(40);AB-HCAHQ(10) (#9*y*) (**a**) and SEM photographs (magnification: 500×) of the carbonized portions for #9*y* (**b**) and the modifier-free TA-MHQ/BPTP(60);4,4′-ODA(40) (#3) (**c**).

Scanning electron microscope (SEM) imaging of the carbonized portion for #9*y* after the burning tests [Figure 5a] revealed a trace of numerous fine bubbles generated during combustion [Figure 5b], whereas no such trace was observed for the pristine PEsI (#3) [Figure 5c]. This suggests that the finely foamed char layers acted as effective heat insulator and thus interrupted the continuous combustion (Figure S2).

Usually, *C*_P_ = 2–5 wt% is required to ensure sufficient flame retardancy, depending on the selected resin [67]. Nonetheless, the AB-HCAHQ-modified system (#9) exhibited excellent flame retardancy (V-0) even at an extremely low *C*_P_ of 0.21 wt%. This suggests that the pristine PEsI inherently had flame-retardant properties to some extent. In other words, even originally flammable PEsI systems have a chance of achieving V-0 in the absence of flame retardants upon structural refinement.

The increase in AB-HCAHQ content from 0 to 5 and 10 mol% (#3, 9*x*, 9*y*, respectively) resulted in gradual deteriorations in several properties, i.e., the decreases in the *T*_g_ and *ε*_b_ and increases in the CTE, *W*_A_, and CHE. This suggests that, if possible, excellent flame retardancy should be achieved without flame retardants (modifiers). Therefore, we investigated the structure–flame retardancy relationship for a series of pure PEsIs to elucidate the main structural factors dominating their flame retardancy.

### 3.5. Strategies to Improve the Flame Retardancy of PEsIs without Flame Retardants

The fundamental concept to significantly reduce the *W*_A_ and CHE of PEsIs while maintaining their excellent flame retardancy is to reduce the content of imide groups (*C*_i_) while avoiding a significant increase in the content of ester connecting groups (*C*_e_). Specifically, the concept is to replace single 1,4-phenylene units in the monomers with naphthylene or multiple *para*-phenylene [*p*-biphenylene (BP), *p*-terphenylene (TP), and *p*-quaterphenylene (QP)] units. Previously, we briefly reported the CTEs of PEsIs obtained from isomeric naphthylene-containing TCDAs (TA-NAs) [27]. In this study, we investigated the properties of a series of isomeric TA-NA-based PEsI films and their flame retardancy in detail.

#### 3.5.1. PEsIs Derived from Isomeric TA-NAs

(a) TA-14NA-based PEsIs

TA-14NA can be viewed as an analog of TA-HQ. The former contains a non-rotatable fused benzene ring as a side group of the HQ unit. The structures of these TCDAs and the properties of TA-14NA-based PEsIs and related systems are presented in Table 4. The TA-14NA monomer showed high polyaddition reactivity with common aromatic diamines and led to homogeneous and viscous solutions of PAAs with sufficiently high *η*_red_ values (>1.0 dL/g). Nonetheless, the PEsI film obtained using 4,4′-ODA (#10) was overly brittle for proper mechanical testing, although the reason for this brittleness was unclear. Despite the flexible structure of 4,4′-ODA, this film (#10) had a very high *T*_g_ (378 °C) exceeding that of its counterpart (TA-HQ/4,4′-ODA, *T*_g_ = 320 °C [21]). These results are probably related to an increased rotational barrier around the central unit in TA-14NA due to an increased rotational sweep volume originating from its fused benzene side group.

The use of *p*-PDA (#11) as another diamine afforded a PEsI film with properties similar to those of its counterpart (TA-HQ/*p*-PDA), namely an extremely high *T*_g_ (423 °C), very low CTE (5.0 ppm K^−1^), very high tensile modulus (6.67 GPa), and extremely low CHE (4.7 ppm/%RH). The observed low CTE and high modulus are closely associated with the significant in-plane chain orientation induced upon thermal imidization of the PAA cast films formed on the substrate, which is unexceptionally observed in PI systems with rigid/linear backbone structures [53,54,55,56,57,58]. This PEsI system (#11, *d* = 16 μm) did not meet the V-0 standard, whereas its counterpart (TA-HQ/*p*-PDA, *d* = 20 μm) passed V-0, corresponding to its discernibly higher thermal and thermo-oxidative stability [*T*_d_^5^ (N_2_) = 481 °C, *T*_d_^5^ (air) = 463 °C [21] than those of #11. Thus, unlike common phenyl substituents, the central fused benzene ring of TA-14NA adversely affected flame retardancy.

The TA-14NA-based systems with other rigid diamines [*m*-TOL (#12), *o*-TOL (#13), APAB (#14), and M-APAB (#15)] also exhibited very high *T*_g_s (>350 °C), high moduli, and ultralow CHEs with somewhat higher CTEs than that of TA-14NA/*p*-PDA. When APAB (#14) was used instead of *p*-PDA, the *W*_A_ significantly decreased from 1.32% to 0.67%, probably because of the concomitant decrease in *C*_i_. However, these PEsIs (#12–15), as well as the *p*-PDA-derived counterpart, were not very tough, probably owing to poor chain entanglement. The *T*_d_^5^ (N_2_) of the TA-14NA-based systems ranged from 440 to 460 °C with a small dependence on the employed diamine. These values were much lower than those of numerous wholly aromatic PIs (*T*_d_^5^ (N_2_) = 520–570 °C by TGA at 10 °C min^−1^, e.g., 567 °C for a home-made PMDA/4,4′-ODA PI film [65]), reflecting the relatively low bonding energy of the ester connecting groups in the PEsIs [47]. Nonetheless, the thermal stability of these PEsIs was sufficiently high for practical applications.

(b) TA-15NA-based PEsIs

Table 5 summarizes the properties of TA-15NA-based PEsIs. Their chain linearity is assumed to be somewhat lower than that of their TA-14NA-based counterparts, as suggested by a crank-shaft-like structure of the TA-15NA-based diimide unit. Correspondingly, the *T*_g_ of the TA-15NA/4,4′-ODA film (#16) was ~40 °C lower (336 °C) than that of the TA-14NA-based counterpart (#10, *T*_g_ = 378 °C). The former (#16) inevitably had a high CTE (51.8 ppm K^−1^) because of the use of a flexible ether-containing diamine (4,4′-ODA), as is often observed for flexible PIs or common polymers [68]. However, despite the use of 4,4′-ODA, which favors chain entanglement, a very tough film was not obtained, as in the case of TA-14NA/4,4′-ODA. 

Compared with TA-14NA/*p*-PDA (#11), TA-15NA/*p*-PDA (#17) resulted in a ~60 °C decreased *T*_g_ (362 °C), somewhat reduced modulus, and increased CTE (21.4 ppm K^−1^), reflecting the crank-shaft-like structure of the latter. The *W*_A_ and CHE were equivalent to those in TA-14NA/*p*-PDA. The TA-15NA/*p*-PDA system (#17, *d* = 30 μm) also exhibited excellent flame retardancy (V-0), in contrast to TA-14NA/*p*-PDA.

The TA-15NA-based systems with other rigid diamines [*m*-TOL (#18), *o*-TOL (#19), APAB (#20), and M-APAB (#21)] also maintained good properties similar to those of TA-15NA/*p*-PDA.

(c) TA-16NA-based PEsIs

The properties of TA-16NA-based PEsIs are listed in Table 6. Even when rigid diamines [*p*-PDA (#23), APAB (#24), and M-APAB (#25)] were used, low CTEs were not obtained, as in the 4,4′-ODA system (#22). These results reflect their highly distorted main-chain structures originating from TA-16NA, which disturb the in-plane chain orientation during thermal imidization. The TA-16NA-based systems also resulted in significantly increased CHEs (13.6–20.9 ppm/%RH), compared with those of other TA-NA-based systems. This is likely associated with the worse in-plane orientation of the former rather than their higher *W*_A_s, as discussed later. The TA-16NA/APAB system (#24) showed somewhat improved film toughness (*ε*_b max_ = 16.5%).

(d) TA-27NA-based PEsIs

Table 7 summarizes the properties of TA-27NA-based PEsI films. TA-27NA has a structure with a slightly higher linearity than TA-16NA, although it is not as linear as TA-14NA. Nonetheless, the TA-27NA/4,4′-ODA film (#26) exhibited a very high *T*_g_ (381 °C) close to that of its TA-14NA-based counterpart (#10). As predicted, the former (#26) showed a high CTE (50.4 ppm K^−1^), similar to other 4,4′-ODA-based counterparts (#10, 16, 22).

The *p*-PDA system (#27) exhibited an extremely high *T*_g_ (404 °C) and relatively low CTE (22.7 ppm K^−1^) equivalent to that of the crank-shaft-type TA-15NA-based counterpart (#17, CTE = 21.4 ppm K^−1^). However, this film (#27) was overly brittle for proper tensile testing. This system (*d* = 15 μm) also exhibited excellent flame retardancy (V-0).

The systems with other rigid diamines [*o*-TOL (#28), APAB (#29), and M-APAB (#30)] showed relatively low CTEs, which were slightly higher than those of the TA-15NA-based counterparts (#19, 20, 21). Unexpectedly, the use of *o*-TOL (#28) discernibly improved film toughness (*ε*_b max_ = 39.9%), whereas no significant toughening was observed for other *o*-TOL-based counterparts (#13, 19, 34). The mode of action for this specific effect is unknown.

(e) TA-26NA-based PEsIs

The properties of TA-26NA-based PEsIs are summarized in Table 8. The 4,4′-ODA system (#31) exhibited a very high *T*_g_ (361 °C) with a *T*_β_ at 208 °C. This film was ductile because of the flexibility of 4,4′-ODA, although the *ε*_b_ was not as high as expected. Moreover, this system (*d* = 22 μm) did not meet V-0.

The use of *p*-PDA (#32) resulted in an extremely high *T*_g_ (418 °C), ultralow CTE (2.9 ppm K^−1^), and the highest *T*_d_^5^ (N_2_) (499 °C) and *T*_d_^5^ (air) (489 °C) among the TA-NA-based systems, which were higher than those of TA-HQ*/p*-PDA [*T*_d_^5^ (N_2_) = 481 °C and *T*_d_^5^ (air) = 463 °C [21]]. The TA-26NA/*p*-PDA system (*d* = 17 μm) also exhibited excellent flame retardancy (V-0), corresponding to its high *T*_d_^5^ values mentioned above. When compared with the fact that TA-26NA/4,4′-ODA (#31) did not meet V-0, the impact of the diamines on the improvement in flame retardancy is ranked as *p*-PDA > 4,4′-ODA. However, this result could not be explained by considering only the difference between the contents of the thermally less stable ester groups (*C*_e_) because the former (#32) has a higher *C*_e_ (15.2 wt%) than the latter (13.1 wt% for #31). 

To explain the above results, we propose the following hypothesis: PEsI main chains highly aligned in the *XY*-direction, corresponding to the low CTEs typical of the TA-26NA/*p*-PDA system, can facilitate the formation of char layers during combustion, which effectively interrupt heat transfer and oxygen permeation necessary for continuous flaming (Figure S2). 

A similar situation was observed when the highly linear structure of TA-HQ/*p*-PDA (Figure 1) with an extremely low CTE (3.2 ppm K^−1^) and high Δ*n*_th_ (0.219) [21] was compared with the distorted structure of the isomeric TA-RC/*p*-PDA (Figure 1) with a high CTE (50.6 ppm K^−1^) and low Δ*n*_th_ (0.036); the former satisfied V-0, whereas the latter did not. A related phenomenon was observed for graphite formation upon heating finally at 3000 °C in an oxygen-free atmosphere; the crystallinity of the resulting graphite was closely associated with the extents of in-plane chain orientation of the wholly aromatic PI films used as the starting materials [69].

The TA-26NA-based systems with other rigid diamines (#33–36) also exhibited relatively low CTEs. In addition, regardless of the diamine, all TA-26NA-based systems exhibited ultralow CHEs (1.0–4.7 ppm/%RH). 

**Scheme 11:**
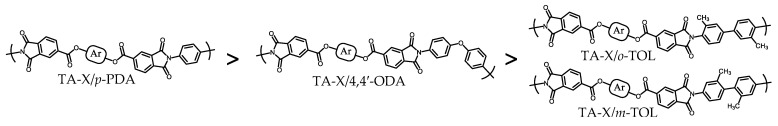
Impact of diamine on the improvement of flame retardancy for TA-X-based PEsIs.

The TA-26NA/*m*-TOL system (#33, *d* = 22 μm), as well as its analogs [TA-HQ/*m*-TOL and TA-HQ/*o*-TOL], did not meet V-0. The results are very likely ascribed to the low thermal stability of the CH_3_ groups therein. Thus, based on the comparison of TA-26NA/*p*-PDA (#32, V-0 passed) and TA-26NA/*m*-TOL (#33, V-0 failed), the impact of the diamines on the improvement of flame retardancy is ranked as *p*-PDA > *m*-TOL. This is supported by the comparison of TA-HQ/*p*-PDA (V-0 passed), TA-HQ/*o*-TOL (V-0 failed), and TA-HQ/*m*-TOL (V-0 failed). In addition, the modification of TA-HQ/*o*-TOL and TA-HQ/*m*-TOL by copolymerization with 4,4′-ODA (25 mol%) dramatically improved their flame retardancy, suggesting that the impact of the diamines on the improvement of flame retardancy is ranked as 4,4′-ODA > *o*-TOL (≈*m*-TOL). The overall order of the impact of diamine is shown in Scheme 11.

(f) Comparative Analysis of the Properties of TA-NA-based PEsIs

**Figure 6:**
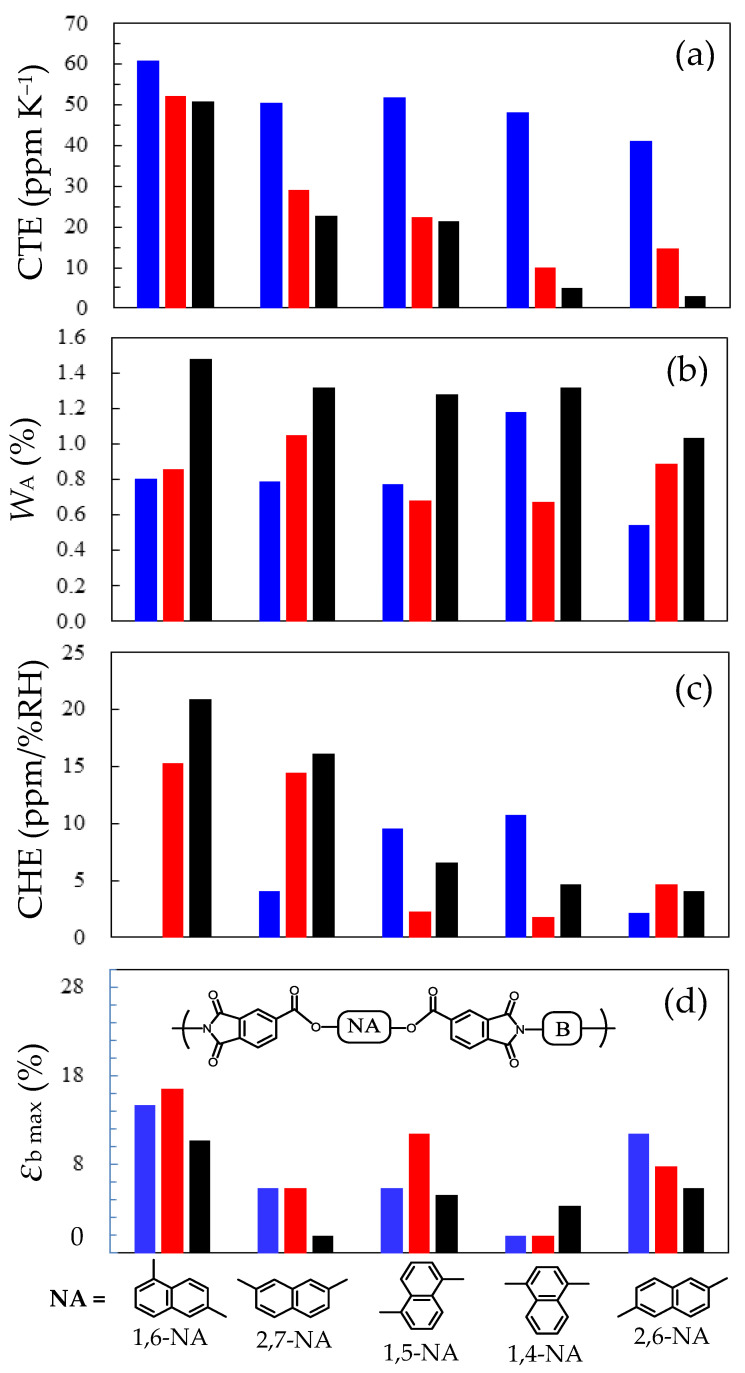
Isomer effects on the properties for the naphthalene-containing PEsIs obtained from different diamines [4,4′-ODA (left, blue bar), APAB (center, red bar), and *p*-PDA (right, black bar)]: (**a**) CTE, (**b**) *W*_A_, (**c**) CHE, and (**d**) *ε*_b max_. (0% for the samples that were too brittle for the tensile testing). The general structure of the PEsIs and the substructures of TA-NAs are inserted in this figure.

Figure 6 compares the properties of isomeric TA-NA-based PEsIs using three selected diamines (4,4′-ODA, *p*-PDA, and APAB). In the abscissa of this figure, the substructures of the isomeric TA-NAs are arranged from left to right in the order of increasing structural linearity (1,6-NA < 2,7-NA < 1,5-NA < 1,4-NA ≤ 2,6-NA).

The isomer effect on the CTE is shown in Figure 6a. As predicted, all isomeric systems using 4,4′-ODA (left, blue bar) had high CTEs. However, the CTE of the 4,4′-ODA-based systems tended to decrease with increasing substructural linearity. This trend was particularly noticeable in the systems using APAB (center, red bar) and *p*-PDA (right, black bar).

Figure 6b shows the isomer effect on the *W*_A_. In many cases, the *W*_A_ decreased depending on the diamine in the order of *p*-PDA > APAB > 4,4′-ODA. The stronger *W*_A_-reducing effect of 4,4′-ODA than that of APAB is probably associated with the lower polarity of the former, as suggested by a lower ratio of the molar polarization (*P*_m_) to molar volume (*V*_m_) for the ether group in 4,4′-ODA (*P*_m_/*V*_m_ = 0.520) than that for the ester group in APAB (0.652) [70]. Although the dependence of the *W*_A_ on the TA-NA structure was less prominent than that of CTE, the *W*_A_ roughly tended to decrease with an increase in the structural linearity of TA-NAs. However, this behavior has no relation to the *C*_i_, which is constant for the isomeric systems. The rough trend observed for the *W*_A_ likely reflects that higher chain linearity favors closer chain stacking, which is unfavorable for water absorption.

As shown in Figure 6c, the CHE clearly decreased with increasing substructural linearity particularly in the APAB- and *p*-PDA-based systems, similar to the effect on the CTE. This behavior implies that the CHE was strongly affected by not only the *W*_A_ but also the extent of in-plane chain orientation [24]; highly plane-oriented films include more chain components aligned in the CHE-measuring (*X*) direction than three-dimensionally random-oriented films (Figure S3). The polymer chains oriented in the *X*-direction usually have strong resistance to the *X*-directional expansion due to water absorption.

Figure 6d shows an isomer effect on the *ε*_b_. No clear trend was observed for *ε*_b_. Furthermore, even the systems using flexible 4,4′-ODA did not show a significant toughening effect, regardless of TA-NA.

Thus, the combinations of TA-26NA and rigid diamines were deemed suitable for simultaneously achieving low CTE, *W*_A_, and CHE. In particular, the TA-26NA/*p*-PDA system (#32) also demonstrated excellent flame retardancy (V-0). However, the issue of toughness improvement remained unresolved.

#### 3.5.2. PEsIs Derived from *para*-Phenylene-containing TCDAs (TA-*p*Ph)

As mentioned previously, to simultaneously achieve low CTE, *W*_A_, CHE, and excellent flame retardancy, a considerable reduction in the *C*_i_ without significantly increasing the *C*_e_ is required. For this purpose, we focused on a series of ester-linked TCDAs containing multiple *p*-phenylene units as alternatives to TA-NAs.

(a) TA-44BP-based PEsIs

Table 9 summarizes the properties of TA-44BP-based PEsI films. TA-44BP showed high polyaddition reactivity with common aromatic diamines and led to homogeneous and viscous solutions of PAAs with sufficiently high *η*_red_ values (high molecular weights).

The 4,4′-ODA system (#37) exhibited a considerably high *T*_g_ (376 °C) with a *T*_β_ (219 °C), in addition to a low *W*_A_ (0.30%) and ultralow CHE (0.23 ppm/%RH). A pronounced toughening effect was observed, unlike the poor effect in the TA-26NA-based counterpart (#31). The TA-44BP/4,4′-ODA film (#37) also had higher *T*_d_^5^ (N_2_) and *T*_d_^5^ (air) than the TA-26NA-based counterpart, probably reflecting the higher thermal and thermo-oxidative stability of the *p*-biphenylene unit in the former than those of the 2,6-naphthylene unit in the latter. However, the former (#37, *d* = 25 μm) did not meet V-0, similar to the latter.

The *p*-PDA system (#38) exhibited excellent combined properties equivalent to those of the TA-26NA-based counterpart (#32), i.e., a DMA-undetectable *T*_g_ [Figure S4a], ultralow CTE, and extremely low CHE. This PEsI (#38, *d* = 21 μm) also exhibited excellent flame retardancy (V-0), corresponding to its increased *T*_d_^5^ values (N_2_ and air), compared with those of the 4,4′-ODA-based counterpart (#37). Based on the comparison of TA-44BP/*p*-PDA (#38, V-0 passed) with TA-44BP/4,4′-ODA (#37, V-0 failed), the impact of the diamines on the improvement of flame retardancy is ranked as *p*-PDA > 4,4′-ODA, as in the aforementioned TA-NA-based systems.

The systems using other rigid diamines (#39–43) had properties similar to those of their TA-26NA-based counterparts and maintained relatively low CTEs.

(b) TA-DMBP-based PEsIs

Table 10 lists the properties of PEsIs based on TA-DMBP, a dimethyl-substituted version of TA-44BP. The *T*_g_ of the 4,4′-ODA system (#44) was ~30 °C lower (347 °C) than that of its non-substituted TA-44BP-based counterpart (#37). The former (#44) also had lower *T*_d_^5^ (N_2_) and *T*_d_^5^ (air) (by ~40 and ~60 °C, respectively) than those of the TA-44BP-based counterpart, owing to the thermally less stable CH_3_ groups of TA-DMBP.

In the *p*-PDA system (#45), the introduction of CH_3_ substituents resulted in a *T*_g_ barely detectable by DMA [Figure S4b] and much lower *T*_d_^5^ (N_2_) and *T*_d_^5^ (air) than those of its CH_3_-free counterpart. Despite the presence of the CH_3_ substituents, this PEsI (#45) achieved V-0, whereas the TA-DMBP/*o*-TOL system (#47) did not, probably owing to the increased CH_3_ content (*C*_m_) of the latter.

In the systems with other rigid diamines (#46–49), similar CH_3_ substituent effects, i.e., decreases in *T*_g_ and *T*_d_^5^ were observed. Thus, the introduction of CH_3_ groups into TA-44BP had almost no positive influence on the target properties of the corresponding systems.

(c) TA-DPBP-based PEsIs

The properties of diphenyl-substituted TA-DPBP-based PEsIs are summarized in Table 11. The 4,4′-ODA system (#50) showed a distinct thermal transition at 222 °C on DMA. This transition probably corresponds to an intensified *β*-transition, which is often observed around 200 °C for TA-*p*Ph-based PEsIs with substituents, rather than to a significantly decreased *T*_g_. This system (#50) also had a high CTE (60.1 ppm K^−1^), as is often observed for flexible PIs and common polymers [68], similarly to its counterparts obtained using other flexible diamines [3,4′-ODA (#51) and BAPS (#52)].

The *p*-PDA system (#53) exhibited a complex DMA curve including a *β*-transition at 235 °C [Figure S5a]. A typical double-transition behavior was observed when this PEsI film was annealed at a higher temperature [Figure S5b]. The relatively low CTE (21.7 ppm K^−1^), low *W*_A_, ultralow CHE, and improved *T*_d_^5^ values of this system (#53) are ascribed to the replacement of the CH_3_ substituents with the thermally more stable phenyl side groups. In addition, this system (*d* = 26 μm) satisfied V-0.

The properties of the systems based on other rigid diamines (#54–58) were approximately equivalent to those of the *p*-PDA-based system (#53), except for *T*_d_^5^ (Table 11).

(d) TA-MTP-based PEsIs

Table 12 summarizes the properties of PEsIs derived from TA-MTP, which contains a CH_3_-substituted *p*-terphenylene (TP) unit. The corresponding substituent-free TCDA was not available because of the difficulty in synthesizing it due to the insufficient solubility of its raw material. The system using 4,4′-ODA (#59) exhibited discernibly higher *T*_g_ and *T*_d_^5^ values than those of its TA-DMBP-based counterpart (#44). In particular, the higher *T*_d_^5^ of the former is due to its decreased *C*_m_. Other properties were equivalent to those of the TA-DMBP-based counterpart (#44).

The use of *p*-PDA (#60) resulted in a higher *T*_g_ and *T*_d_^5^ values than those of the TA-DMBP-based counterpart (#45). The former (*d* = 25 μm) also exhibited excellent flame retardancy (V-0) without the negative impact of the CH_3_ substituent. Other properties, including ultralow CTE and CHE, were equivalent to those of its counterpart [TA-DMBP/*p*-PDA (#45)].

Similarly, the systems with other rigid diamines (#61–65) tended to show higher *T*_g_ and *T*_d_^5^ values than those of their TA-DMBP-based counterparts. A feature was observed in the systems derived from *m*-TOL (#61) and TFMB (#65); their film toughness was significantly improved, despite almost non-promising chain entanglement due to their rigid and linear main-chain structures.

(e) TA-PTP-based PEsIs

The properties of PEsIs derived from the phenyl-substituted TA-PTP are listed in Table 13. The system with 4,4′-ODA (#66) exhibited appreciably improved *T*_d_^5^ values, compared with those of its TA-MTP-based counterpart (#59) because of the replacement of the CH_3_ substituents with the thermally/thermo-oxidatively more stable phenyl side groups. Another prominent feature was the significantly improved film toughness. This PEsI (#66) showed double thermal transition behavior with a distinct *T*_g_ (337 °C) and *T*_β_ (197 °C). The latter tended to be intensified, particularly when TA-PTP was combined with flexible diamines, as typically observed for the systems with 3,4′-ODA (#67) and BAPS (#68); the thermal transitions at 200–235 °C in these systems probably correspond to intensified *β*-transitions rather than to significantly decreased *T*_g_s.

The TA-PTP/*p*-PDA (#69, *d* = 30 μm) and TA-PTP/*o*-TOL (#71, *d* = 18 μm) systems were compatible with V-0. The excellent flame retardancy of the latter was probably caused by the “diluted” *C*_m_ in the repeating unit as a result of the increased aromatic content due to the phenyl substituent. The combinations of TA-PTP and rigid diamines [*p*-PDA (#69), *m*-TOL (#70), *o*-TOL (#71), APAB (#72), and M-APAB (#73)], despite their almost non-promising chain entanglement, exhibited significantly improved film toughness, compared with those of their TA-MTP-based counterparts, while maintaining relatively low CTEs (16–38 ppm K^−1^). The toughening effect was particularly prominent in the system with TFMB (#74). A possible mechanism for this unexpected effect will be mentioned later.

(f) TA-DMQP-based PEsIs

The properties of PEsIs derived from TA-DMQP, including a dimethyl-substituted *p*- quaterphenylene (QP) unit, are listed in Table 14. The system with 4,4′-ODA (#75) exhibited a somewhat reduced CTE (46.7 ppm K^−1^) and *W*_A_ (0.47%), compared with those of the TA-MTP-based counterpart (#59), probably owing to the extended QP moiety and the decreased *C*_i_, respectively. Other properties were equivalent to those of the TA-MTP-based counterpart. The modification of the systems using rigid diamines (*p*-PDA and APAB) by copolymerization with 4,4′-ODA (#76, 77) resulted in relatively balanced properties.

(g) TA-DPQP-based PEsIs

Table 15 summarizes the properties of diphenyl-substituted TA-DPQP-based PEsIs. The 4,4′-ODA system (#78) afforded a highly tough film (*ε*_b max_ = 51.5%) and exhibited a discernibly reduced CTE and slightly decreased *W*_A_ with other equivalent properties, compared with those of its TA-PTP-based counterpart (#66).

The *p*-PDA-based system (#79) exhibited satisfactory film toughness (*ε*_b max_ = 36.2%), despite almost non-promising chain entanglement due to its rigid/linear main-chain structure. In addition, this system showed an ultralow CHE and lower CTE and *W*_A_ values than those of its TA-PTP-based counterpart (#69). This PEsI also had excellent flame retardancy (V-0).

The systems with other rigid diamines (#80–83) showed similar properties to those of their TA-PTP-based counterparts (#70–73).

The TA-DMQP/*p*-PDA system was modified by random copolymerization with TA-44BP (30 mol%). This approach (#84) resulted in an increased tensile modulus, although no clear CTE-reducing effect was observed. Sequence-controlled copolymerization [71], which has a certain effect on reducing the CTE and improving the film toughness in some cases, was applied to our system. This approach (#85) somewhat improved the *ε*_b max_, compared with that of the random copolymer (#84); however, no discernible CTE-reducing effect was observed.

An increase in the TA-44BP content to 50 mol% (#86) resulted in a slight reduction in CTE. This copolymer was further modified by random copolymerization with 4,4′-ODA (20 mol%) (#87) to improve film toughness. However, this approach was less effective.

The above copolymers (#84–87, *d* = 24–31 μm), as well as the homo TA-DPQP/*p*-PDA system (#79, *d* = 28 μm), had excellent flame retardancy (V-0). For these copolymers, the burning time during the UL-94V tests was considerably shorter than for the corresponding homo system (#79), as observed in the TA-44BP/*p*-PDA system (#38). Thus, TA-44BP was a useful modifier.

PEsIs derived from a series of TA-*p*Phs, except for TA-DPBP/BAPS (#52) and TA-DPBP/TFMB (#58), were insoluble in various solvents.

(h) Comparative Analysis of the Properties of TA-*p*Ph-based PEsIs

**Figure 7:**
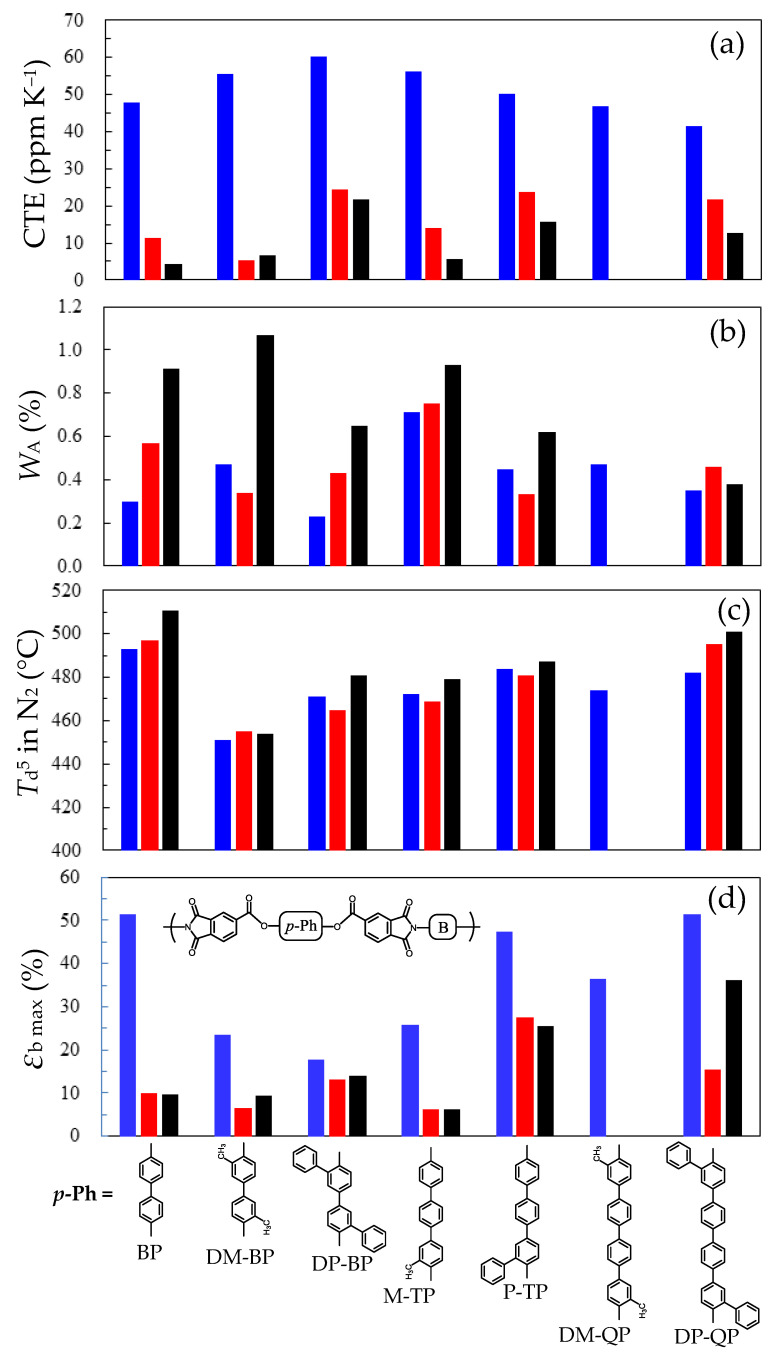
Comparisons of the properties for the TA-*p*Ph-based PEsIs obtained from different diamines [4,4′-ODA (left, blue bar), APAB (center, red bar), and *p*-PDA (right, black bar)]: (**a**) CTE, (**b**) *W*_A_, (**c**) *T*_d_^5^ (N_2_), and (**d**) *ε*_b max_.

Figure 7 compares the properties of PEsIs derived from a serious of TA-*p*Phs and three selected diamines (4,4′-ODA, *p*-PDA, and APAB). In the abscissa of this figure, the central substructures of TA-*p*Ph are arranged from left to right in the order of increasing *p*-phenylene unit length (*L*_pPh_; *p*-BP < *p*-TP < *p*-QP).

The effect of the diamine structure on the CTE is shown in Figure 7a. The CTE decreased in the order of 4,4′-ODA (blue bar) >> APAB (red bar) > *p*-PDA (black bar), in agreement with the trend in structural rigidity/linearity of the diamines. However, the dependence of the CTE on *L*_pPh_ was not monotonous; the CTE tended to increase upon going from BP to DP-BP, and to decrease upon going from DP-BP to DP-QP (see the blue bars as the typical change). Thus, a significant extension of the central *p*-phenylene structures (e.g., TA-DPQP) was advantageous for lowering the CTE.

Figure 7b shows the dependence of *W*_A_ on the diamine structure and *L*_pPh_. The *W*_A_ tended to decrease depending on the diamine in the order of *p*-PDA > APAB > 4,4′-ODA, as in the case of TA-NA-based systems [Figure 6b]. Moreover, the *W*_A_ gradually decreased with increasing *L*_pPh_ (typically see the black bars), reflecting the decrease in *C*_i_.

The dependence of *T*_d_^5^ (N_2_) on the diamine structure and *L*_pPh_ is shown in Figure 7c. The *T*_d_^5^ (N_2_) roughly increased in the order of APAB < 4,4′-ODA < *p*-PDA, corresponding to the thermal stability order of the connecting groups (ester < ether) [47]. Except for the TA-44BP system, the *T*_d_^5^ (N_2_) gradually increased with increasing *L*_pPh_ while zigzagging with a small downturn in CH_3_-substituted systems and a small upturn in phenyl (Ph)-substituted systems. The gradual increase in *T*_d_^5^ (N_2_) results from the decreased content of the thermally less stable ester groups.

Figure 7d shows the dependence of *ε*_b_ on the diamine structure and *L*_pPh_. The *ε*_b max_ decreased in the order of 4,4′-ODA >> APAB ≈ *p*-PDA, corresponding to their rotational flexibility, which is closely related to chain entanglement. The lower film toughness of APAB-based systems probably reflects the limited internal rotation at the ester linkage in APAB, in contrast to the free rotation at the ether linkage in 4,4′-ODA. When the central unit was changed from DP-BP to DP-QP, the film toughness was improved, particularly for the phenyl-substituted systems based on TA-PTP and TA-DPQP.

(i) Superiority of the Substituents in TA-*p*Phs

**Figure 8:**
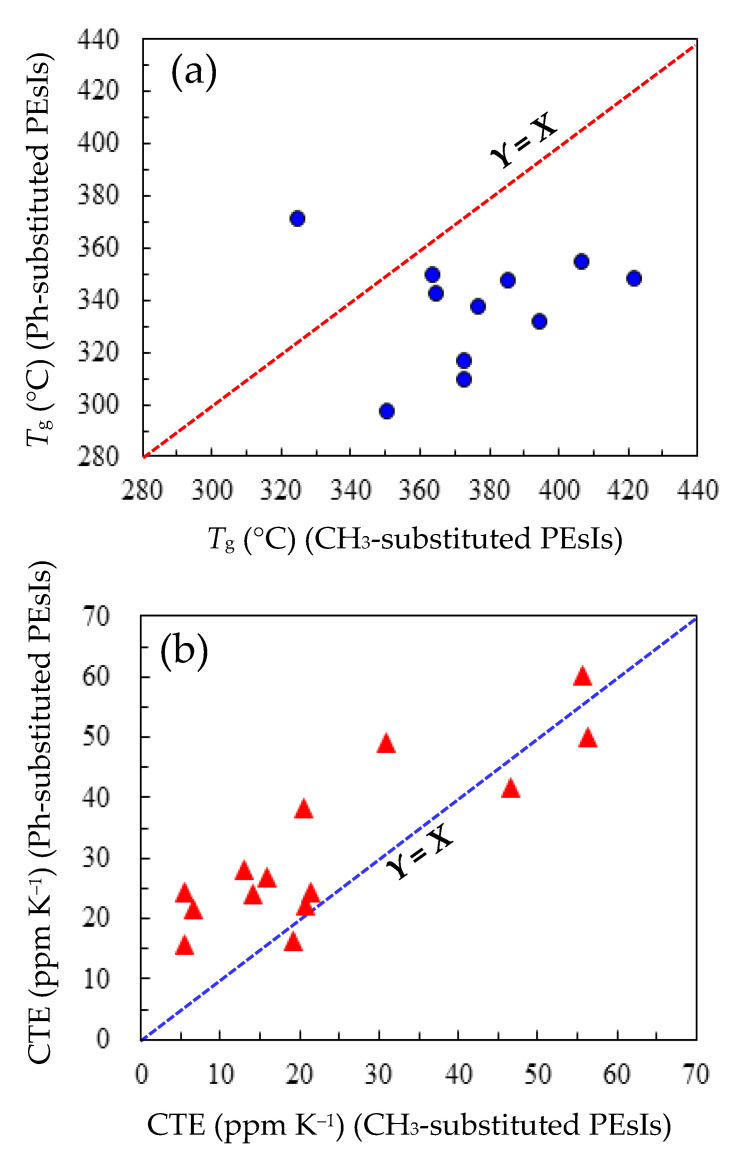

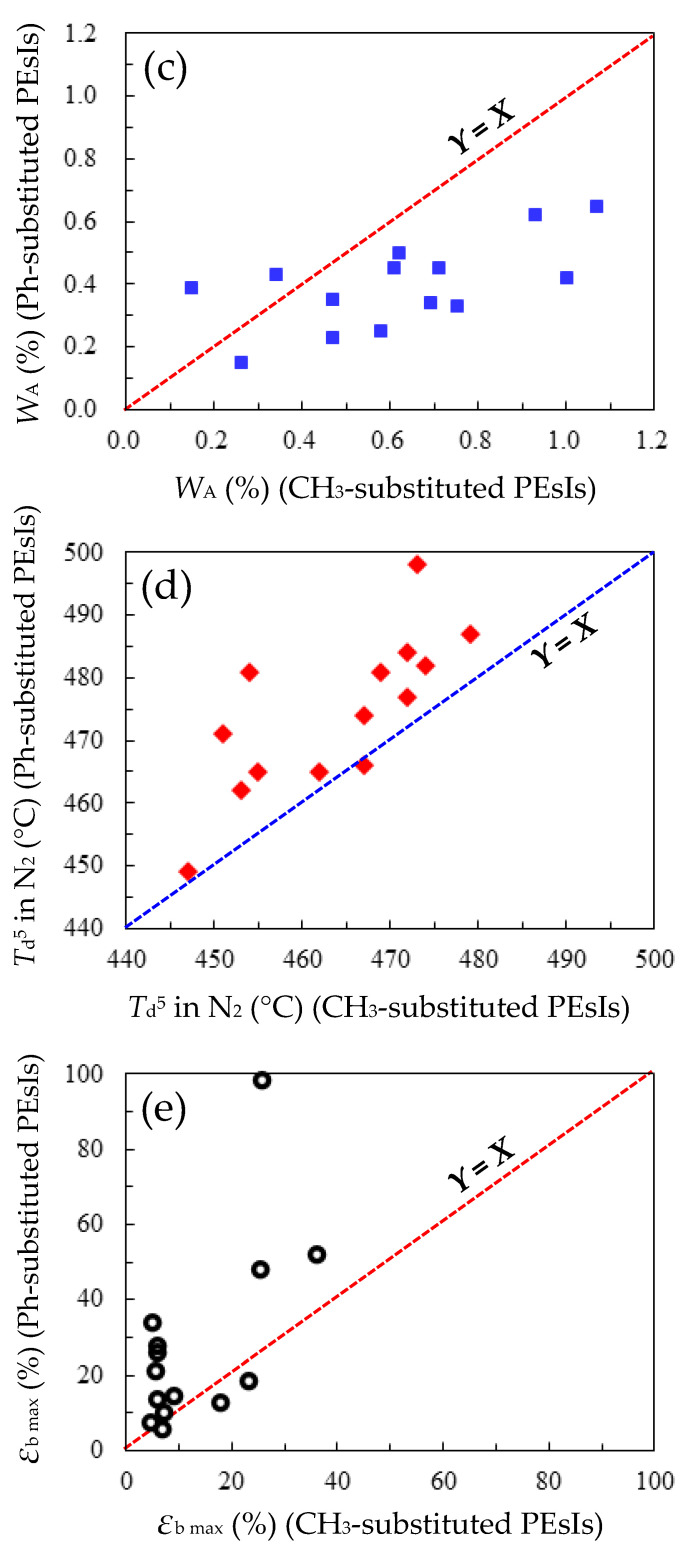
The superiority of the substituents of TA-*p*Phs (phenyl and methyl groups) in the properties: (**a**) *T*_g_, (**b**) CTE, (**c**) *W*_A_, (**d**) *T*_d_^5^ (N_2_), and (**e**) *ε*_b max_. The dashed lines represent the *Y* = *X* relationship.

Figure 8a shows the relationship between the *T*_g_s of Ph- (*Y*-axis) and CH_3_-substituted (*X*-axis) PEsI systems with the identical *L*_pPh_ and diamines. In this figure, most of the datapoints are located below the *Y* = *X* line, indicating that the Ph-substituted systems usually have lower *T*_g_s than the corresponding CH_3_-substituted systems. This result probably reflects the interruption of the close chain stacking by the bulky phenyl side groups.

A similar comparison for the CTE is shown in Figure 8b. In this figure, most of the datapoints are located above the *Y* = *X* line, indicating that the Ph-substituted systems usually have higher CTEs than the corresponding CH_3_-substituted systems. Based on the fact that the phenyl group is considerably bulkier than the CH_3_ group, this result does not conflict with our previous findings [57]; the introduction of excessive substituents hindered in-plane chain orientation during thermal imidization, which suggests the importance of close inter-chain contact for in-plane orientation enhancement.

On the other hand, the superiority of the Ph-substituted systems for *W*_A_ can be observed in Figure 8c; they usually exhibited lower *W*_A_s than the corresponding CH_3_-substituted systems, corresponding to the lower *C*_i_s of the former.

In addition, the *T*_d_^5^ (N_2_) values of the Ph-substituted systems were always higher those of their CH_3_-substituted counterparts [Figure 8d], indicating that the local thermal stability of phenyl side groups considerably exceeded that of CH_3_ groups.

Finally, Figure 8e demonstrates that the Ph-substituted systems are superior to their CH_3_-substituted counterparts in terms of film toughness. The toughening mode of action is discussed later.

#### 3.5.3. Comprehensive Structure–Property Relationships for TA-NA- and TA-*p*Ph-based PEsIs

(a) *W*_A_ and CHE

**Figure 9:**
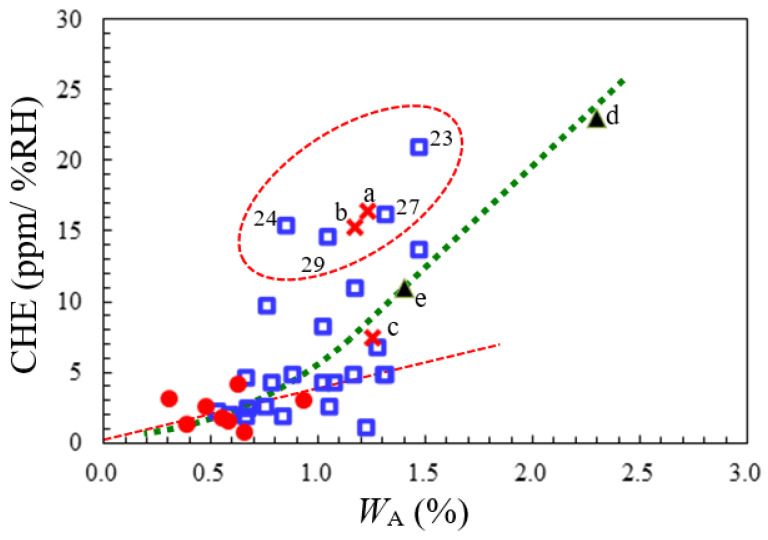
Correlation of CHE and *W*_A_ for PEsI or PI films: (●) TA-*p*Ph-based PEsIs, (□) TA-NA-based PEsIs, (×) conventional PEsIs [(a) TA-RC/*o*-TOL, (b) TA-RC/*m*-TOL, and (c) TA-HQ/*p*-PDA], and (▲) commercial PI films [(d) KAPTON^®^ H [18] and (e) UPILEX^®^-S [20]].

Figure 9 presents the CHE–*W*_A_ correlation for TA-NA- and TA-*p*Ph-based systems. Except for the datapoints surrounded by a red-dashed oval, the CHE exhibited a roughly linear decrease with decreasing *W*_A_, although a certain datapoint scatter was observed. The datapoints located significantly above the fitting curve (green-dashed line) belong to the non-low-CTE PEsIs with highly distorted main-chain structures, e.g., the TA-16NA- (#23, 24), TA-27NA- (#27, 29), and TA-RC-based systems (*a*, *b*). Hence, the significantly increased CHEs of these systems are probably associated with their poor in-plane orientation (i.e., almost 3D-random orientation) [24], as shown in Figure S3. The datapoints for TA-*p*Ph-based systems (●) were fitted to another curve (red-dashed line) with a slope smaller than that of the fitting curve for the whole dataset (green-dashed line). This behavior is ascribed to the higher expansion resistance to water absorption in the TA-*p*Ph-based systems, which probably reflects their high extents of in-plane orientation.

(b) Film Toughness

**Figure 10:**
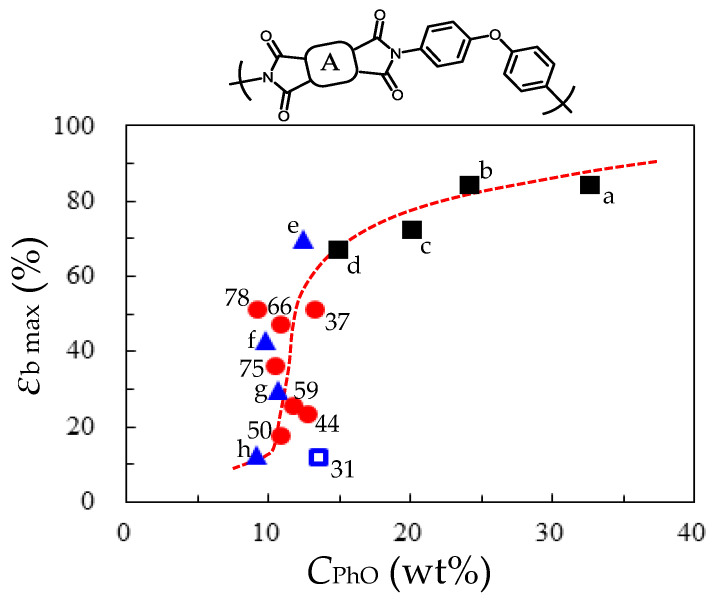
Impact of the 1,4-phenylene ether group (–Ph–O–) content (*C*_PhO_) in the repeating units on the elongation at break (*ε*_b max_) for the PI and PEsI films obtained from 4,4′-ODA and various TCDAs: (a) hydroquinone diphthalic anhydride (HQDA), (b) PMDA, (c) *s*-BPDA, (d) TA-HQ, (e) TA-HPHB, (f) TA-44BPHB, (g) TA-HQHB, (h) TA-26NAHNA. (■) Conventional PIs, (▲) previously reported PEsIs [24,27], and (□,●) PEsIs examined in this study (#31, 37, 44, 50, 59, 66, 75, and 78).

Figure 10 shows the impact of the content of the 1,4-phenylene ether (–Ph–O–) unit, which plays an important role in chain entanglement, on film toughness (*ε*_b max_). Conventional 4,4′-ODA-derived PIs (■, *a–d*) had significantly high *ε*_b max_ values, reflecting their high –Ph–O– contents (*C*_PhO_). In the 4,4′-ODA-based conventional PEsIs (▲, *e–g*), the *ε*_b_ decreased with a decrease in *C*_PhO_ by the structural extension via ester groups and abruptly reduced below *C*_PhO_ ≈ 12 wt%, as shown in the fitting curve (red-dashed line). The PEsI derived from a longitudinally significantly extended TCDA (*N*_Ar_ = 8, ▲, *h*) with a significantly decreased *C*_PhO_ (9.1 wt%) did not afford a highly tough film (*ε*_b max_ = 12.5% [27]), despite the use of flexible 4,4′-ODA. This result suggests that the embrittlement issue at low *C*_PhO_ is difficult to avoid.

The TA-26NA/4,4′-ODA system (**□**, #31) had an unexpectedly low toughness (*ε*_b max_ = 11.5%), even though its *C*_PhO_ (13.7 wt%) was not overly low. A similar situation was observed for other TA-NA/4,4′-ODA systems.

In contrast, some TA-*p*Ph-based PEsIs exhibited a discernible upward shift from the fitting curve, e.g., the TA-DPQP/4,4′-ODA system (#78) maintained an extremely high *ε*_b max_ exceeding 50%, despite its low *C*_PhO_ (9.2 wt%). However, this result does not mean that pronounced toughening is always observed when TA-DPQP is used.

**Figure 11:**
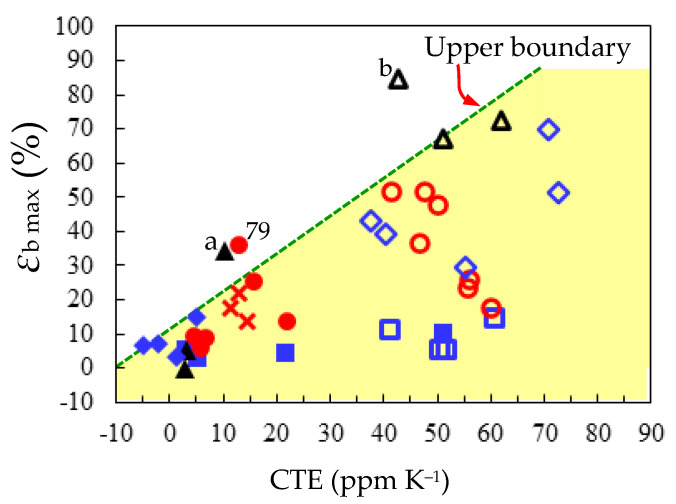
Relationship between CTE and *ε*_b max_ for the homo PEsIs obtained from *p*-PDA (closed symbols) or 4,4′-ODA (open symbols) with various TCDAs: (●,○) TA-*p*Ph, (■,□) TA-NA, (♦,◊) previously reported ester-linked TCDAs [21]. The data of the TA-DPQP-based copolymers (×) and conventional PIs [a: *s*-BPDA/*p*-PDA (▲), b: PMDA/4,4′-ODA) (Δ)] are also plotted for comparisons.

Figure 11 exhibits the relationship between the film toughness (*ε*_b max_) and CTE for TA-NA- and TA-*p*Ph-based PEsIs, revealing that low-CTE systems tend to afford low-*ε*_b_ films because their rigid/linear main-chain structures do not favor chain entanglement. Contrarily, numerous datapoints for high-*ε*_b_ systems were found in the high-CTE range. Moreover, a rough separation was observed between the *p*-PDA-derived low-CTE systems (<~20 ppm K^−1^, closed symbols: ●, ■, ♦, ▲) and the 4,4′-ODA-derived high-CTE systems (>~40 ppm K^−1^, open symbols: ○, □, ◊, Δ).

A clear upper boundary (dashed line) can also be observed in Figure 11. This boundary represents the large difficulty in overcoming the general CTE–*ε*_b_ relationship. The majority of the datapoints of TA-*p*Ph-based systems (●,**○**) are positioned relatively close to the upper boundary, whereas those of TA-NA-based systems (**■,□**) are located far below this boundary. Thus, the superiority of the former in terms of film toughness was confirmed again.

*s*-BPDA/*p*-PDA (*a*) and PMDA/4,4′-ODA (*b*) were among the few located above the upper boundary (Figure 11). In particular, the toughening mode of action for *s*-BPDA/*p*-PDA, which features rigid/linear main chains without ether linkages, is unknown. The TA-DPQP/*p*-PDA system (#79) examined herein also exhibited a similar unexpected toughening effect. To reasonably explain this mode of action, we identified the primary factors influencing film toughness.

Numerous highly ductile PI films show typical stress (S)–strain (S) curves without a yield point [Figure S6a] in stretching tests, unlike common thermoplastic linear polymers. The toughness of the specimens, which is often represented by the fracture energy (*E*_fr_, the area below the S–S curve), is generally affected by several factors [72]. A simplified fracture model for linear polymer systems assumes that *E*_fr_ is closely related to the energy required to pull out a polymer chain from a group of entangled chains [73,74,75]. In highly entangled systems [Figure S6(1)], a higher energy is required to complete the chain pull-out during untangling, corresponding to a higher *E*_fr_; consequently, ductile fracture with a high *ε*_b_ is observed [Figure S6a]. Conversely, in poorly entangled systems with overly rigid/linear main chains [Figure S6(2)] or overly low molecular weights [Figure S6(3)], the chain pull-out requires lower energy, and brittle fracture with a low *ε*_b_ is therefore observed [Figure S6b]. However, even in considerably entangled systems with sufficient chain flexibility and molecular weights, stress concentration, which often generates crazes, micro-voids, and bond cleavage, can be responsible for fracture at an early stage of stretching. Based on this consideration, we assumed that two independent factors are involved in toughening, namely the suppression of stress concentration and enhancement of chain entanglement. This suggests that, if stress concentration can be reduced, there is a chance of improving film toughness to some extent even when sufficient chain entanglement is not available.

To explain the unexpectedly increased film toughness in the TA-*p*Ph-based systems [typically, TA-DPQP/*p*-PDA (#79)], we propose a possible mode of action: the toughening is ascribed to the combined effect of the longitudinally extended *p*-QP unit (dilution of *C*_i_) and the bulky phenyl side groups, which effectively interrupted close inter-chain stacking and weakened intermolecular forces, including the dipole–dipole interactions between the imide C=O groups [76,77] and charge–transfer interactions [44]. This condition is probably sufficient for the occurrence of chain slippage [78,79] during stretching and the resulting decrease in stress concentration. A similar beneficial effect of weakening intermolecular forces on film toughness was observed for benzoxazole-containing PIs with controlled *C*_i_ [39,47].

(c) Flame Retardancy

**Figure 12:**
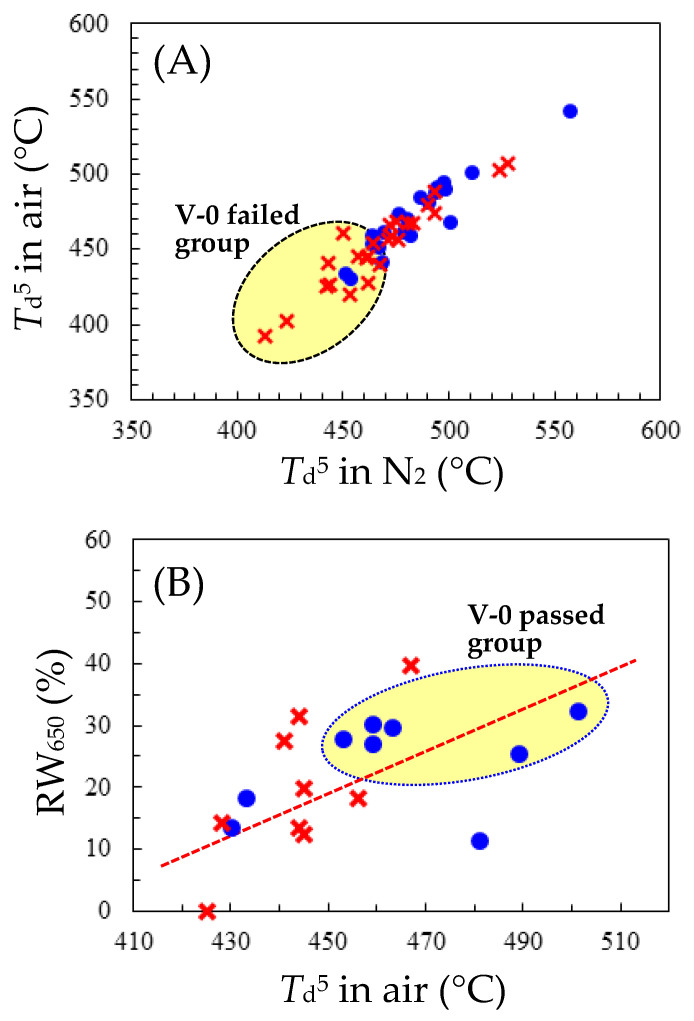

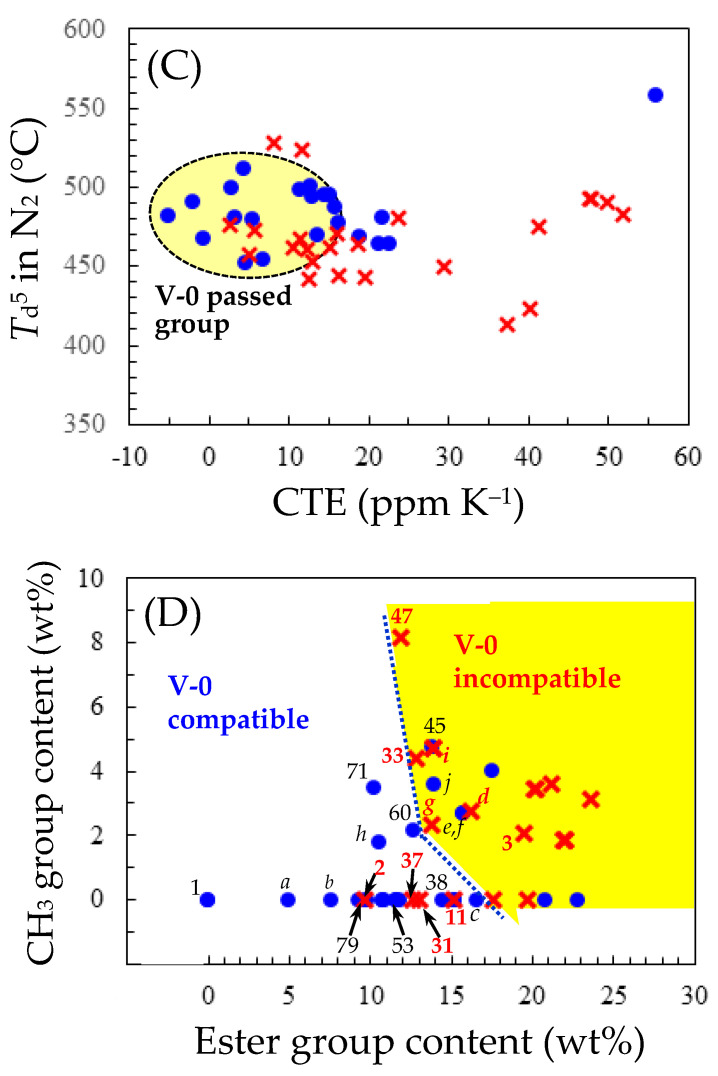
Relationships between the following factors and their impacts on flame retardancy: (**A**) *T*_d_^5^ (N_2_) and *T*_d_^5^ (air), (**B**) residual weights at 650 °C in an air atmosphere (RW_650_) and *T*_d_^5^ (air), (**C**) *T*_d_^5^ (N_2_) and CTE, and (**D**) the contents of methyl and ester groups in various PEsIs: (*a*) *s*-BPDA(70);TA-44BP(30)/4,4′-ODA, (*b*) *s*-BPDA(50);TA-44BP(50)/4,4′-ODA, (*c*) TA-HQ/*p*-PDA, (*d*) TA-MHQ/*p*-PDA, (*e*) TA-MeOHQ/*p*-PDA, (*f*) PMDA/AB-MeOHQ, (*g*) *s*-BPDA/AB-MeOHQ, (*h*) *s*-BPDA/AB-MeOHQ(70);4,4′-ODA(30), (*i*) TA-HQ/*o*-TOL, (*j*) TA-HQ/*o*-TOL(75);4,4′-ODA(25). (●) V-0-compatible and (×) incompatible systems.

Figure 12A presents the correlation among the *T*_d_^5^ (N_2_), *T*_d_^5^ (air), and V-0 compatibility for the PEsIs examined herein and related systems. A good linear relationship between *T*_d_^5^ (N_2_) and *T*_d_^5^ (air) was observed, indicating a strong correlation between thermal and thermo-oxidative stability. Numerous datapoints (enclosed by a blue-dashed oval) in the low *T*_d_^5^ (N_2_) range (<~470 °C) correspond to the systems that did not meet V-0 (**×**). Thus, the systems with low *T*_d_^5^ tended to be easily flammable, probably because they easily generate combustible gases via fragmentation due to bond cleavage.

Figure 12B shows the relationship of *T*_d_^5^ (air), residual weight at 650 °C (RW_650_) in air, and V-0 compatibility. A thermal decomposition mechanism of aromatic PIs in an air atmosphere is proposed [80]. The RW_650_ (air) determined by TGA is assumed to be closely associated with the char layer formability during actual combustion. Indeed, numerous datapoints (enclosed by a blue-dashed oval) located in the high *T*_d_^5^ (air) range (>~450 °C) and the high RW range (>~25%) corresponded to the systems with excellent flame retardancy (V-0).

The relationship of the CTE, *T*_d_^5^ (N_2_), and V-0 compatibility is shown in Figure 12C. In the relatively low CTE range (<~40 ppm K^−1^), a rough negative correlation between the CTE and *T*_d_^5^ (N_2_) was observed. Numerous datapoints corresponding to the V-0-compatible systems (●) were concentrated in the very low CTE range (<~15 ppm K^−1^) and high *T*_d_^5^ (N_2_) range (>~450 °C). In other words, the low-CTE PEsIs tended to have excellent flame retardancy. This result suggests that higher extents of in-plane orientation in low-CTE PEsIs facilitate the formation of char layers, which often have a heat/oxygen-blocking effect, during actual combustion.

Figure 12D exhibits the impact of PEsI composition (contents of ester and CH_3_ groups) on V-0 compatibility. The datapoint at the origin corresponds to wholly aromatic PIs (V-0), such as *s*-BPDA/4,4′-ODA (#1). Numerous datapoints (●) located at low *C*_e_ and *C*_m_ correspond to the V-0-compatible systems, such as CH_3_-free *s*-BPDA;TA-44BP/4,4′-ODA copolymers (*a*, *b*). The TA-DPQP/*p*-PDA system (#79) with a further increased *C*_e_ was also compatible with V-0, as were TA-DPBP/*p*-PDA (#53), TA-44BP/*p*-PDA (#38), and TA-HQ/*p*-PDA (*c*). Despite the presence of CH_3_ groups (<3.5 wt%), TA-PTP/*o*-TOL (#71) maintained excellent flame retardancy (V-0), as did TA-MTP/*p*-PDA (#60) and *s*-BPDA/AB-MeOHQ(70);4,4′-ODA(30) (*h*). In contrast, the flame retardancy tended to deteriorate at higher *C*_e_ and *C*_m_, specifically for TA-MHQ/*p*-PDA (*d*) and TA-MHQ/BPTP(60);4,4′-ODA(40) (#3). Thus, a rough, non-clear-cut boundary between the V-0-compatible and incompatible areas was observed (dashed line).

The presence of both ester and ether groups [e.g., in TA-26NA/4,4′-ODA (#31) and TA-44BP/4,4′-ODA (#37)] resulted in the discernible deterioration of flame retardancy (V-0 failed) in some cases, as revealed by the comparison of TA-26NA/4,4′-ODA (#31, V-0 failed) with TA-26NA/*p*-PDA (#32, V-0 passed) and that of TA-44BP/4,4′-ODA (#37, V-0 failed) with TA-44BP/*p*-PDA (#38, V-0 passed). However, it should be noted that the impact of ether groups on V-0 compatibility is not reflected in Figure 12D.

The number of datapoints corresponding to V-0-incompatible systems (**×**) abruptly increased when *C*_m_ exceeded ~2 wt% and significantly increased above C_e_ ≈ 13 wt%. This result indicates that *C*_m_ has a markedly stronger impact on flame retardancy deterioration than *C*_e_. Thus, the “local” flame retardancy of substituents and connecting group is ranked as CH_3_, OCH_3_ < ester (COO).

The flame retardancy was individually compared to explore the structural factors. TA-HQ/*p*-PDA (*c*) passed V-0, whereas its CH_3_-substituted counterpart [TA-MHQ/*p*-PDA (*d*)] did not. This suggests that the CH_3_ substituent adversely affected flame retardancy. However, when the CH_3_ group was replaced with the OCH_3_ group, the flame retardancy of the resulting TA-MeOHQ/*p*-PDA (*e*) was recovered (V-0 passed). Similarly, its MeO-containing analog, PMDA/AB-MeOHQ (*f*), was compatible with V-0. Thus, the local flame retardancy of substituents is ranked as CH_3_ < OCH_3_ group, although the underlying reason is not clear.

However, the OCH_3_ substituent was not always effective in improving flame retardancy, as suggested by the comparison of the two MeO-containing analogs, PMDA/AB-MeOHQ (*f*) (V-0 passed) and *s*-BPDA/AB-MeOHQ (*g*) (V-0 failed). The V-0 incompatibility of the latter is likely related to a decrease in *C*_i_ upon changing the TCDA from PMDA to *s*-BPDA. The modification of *s*-BPDA/AB-MeOHQ (V-0 failed) (*g*) by copolymerization with 4,4′-ODA (30 mol%) (*h*) resulted in recovered flame retardancy (V-0 passed), as shown in Figure 12D. This is probably because of the concomitant “dilution” of the OCH_3_ and ester groups by copolymerization with 4,4′-ODA. Therefore, the local flame retardancy increases in the order of OCH_3_ < COO < ether (–O–).

A similar effect of copolymerization with 4,4′-ODA was observed; TA-HQ/*o*-TOL (*i*) did not meet V-0, whereas the corresponding TA-HQ/*o*-TOL(75);4,4′-ODA(25) copolymer (*j*) did, probably owing to the dilution of the CH_3_ groups of *o*-TOL by 4,4′-ODA. Thus, the local flame retardancy is ranked as CH_3_ < –O–.

Overall, local flame retardancy increases in the order of CH_3_ < OCH_3_ < COO < –O– < phenyl, 1,4-phenylene, or benzimide groups.

**Figure 13:**
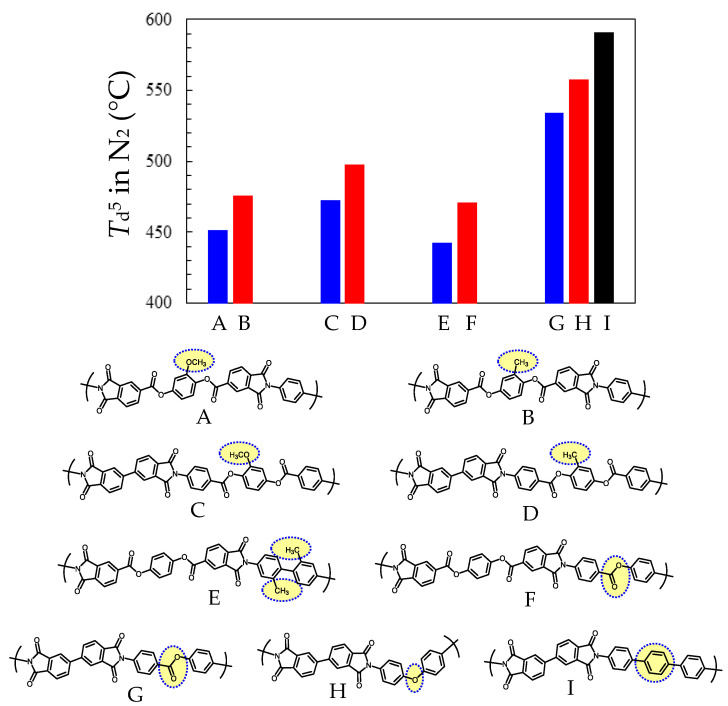
Relative thermal stability of the side groups (–OCH_3_ and –CH_3_ groups) and connecting groups (ester and 1,4-phenylene groups), estimated by the comparisons of the *T*_d_^5^ (N_2_) values of the related PEsI and PI systems: (A) TA-MeOHQ/*p*-PDA [22], (B) TA-MHQ/*p*-PDA [22], (C) *s*-BPDA/AB-MeOHQ [25], (D) *s*-BPDA/AB-MHQ) [25], (E) TA-HQ/*m*-TOL, (F) TA-HQ/APAB [21], (G) s-BPDA/APAB [21], (H) *s*-BPDA/4,4′-ODA [65], (I) *s*-BPDA/4,4″-*p*-terphenylenediamine) [47]. The structures of the related systems (A–I) are shown at the bottom of this figure.

This sequence was compared with that of the *T*_d_^5^ (N_2_)-based local thermal stability of substituents, as shown in Figure 13 (see related PEsI structures at the bottom of this figure); when comparing the *T*_d_^5^ (N_2_) values of the blue bar-A and red bar-B, as well as those of the blue bar-C and red bar-D, the *T*_d_^5^ (N_2_)-based local thermal stability of substituents is ranked as OCH_3_ < CH_3_. From a similar comparison of bar-E and -F, the sequence is estimated as CH_3_ < COO. Additionally, the comparisons of bar-G, -H, and -I show the order of COO < –O– < 1,4-phenylene. Overall, the sequence of local thermal stability is concluded as OCH_3_ < CH_3_ < COO < –O– < 1,4-phenylene group. This was consistent with that of local flame retardancy, except for one part (CH_3_ < OCH_3_). Thus, the close relationship between local flame retardancy and *T*_d_^5^ (N_2_)-based local thermal stability was confirmed again.

**Figure 14:**
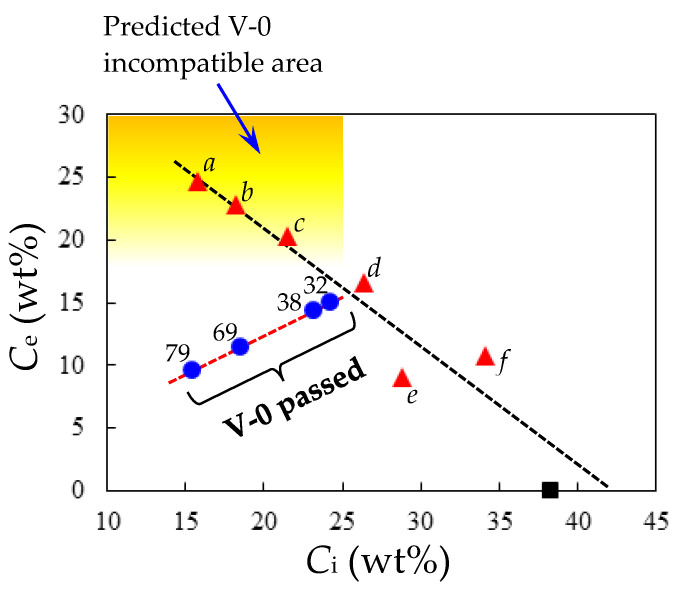
Relationship between the contents of imide (*C*_i_) and ester groups (*C*_e_). (■) *s*-BPDA/*p*-PDA polyimide, (▲) the CH_3_ and OCH_3_-free conventional PEsIs consisting of multiple single aromatic rings *para*-linked via ester groups [(*a*) TA-HQHB/APAB, (*b*) TA-HQHB/*p*-PDA, (*c*) TA-HPHB/*p*-PDA, (*d*) TA-HQ/*p*-PDA, (*e*) *s*-BPDA/APAB, (*f*) PMDA/APAB], and (●) the PEsIs examined in this study: TA-26NA/*p*-PDA (#32), TA-44BP/*p*-PDA (#38), TA-PTP/*p*-PDA (#69), and TA-DPQP/*p*-PDA (#79).

The above results suggest that highly flame-retardant PEsIs can be realized by decreasing *C*_e_ as much as possible, while avoiding the introduction of alkyl and alkoxy groups. However, our strategy for reducing *W*_A_ and CHE by decreasing *C*_i_ was difficult to implement in cases where single aromatic rings were connected via ester groups (Scheme 1), as a concomitant significant increase in *C*_e_ was not easy to avoid. This situation is illustrated in Figure 14; for conventional PEsIs and PIs (▲), *C*_e_ increased almost linearly with decreasing *C*_i_ and reached a predicted V-0-incompatible area with a non-clear-cut boundary.

Conversely, the present approach using TA-*p*Phs, which comprise multiple *p*-phenylene units, allowed us to avoid the above-mentioned dilemma, and the corresponding datapoints (●) gradually moved away from the V-0-incompatible area with decreasing *C*_i_ (Figure 14). In particular, the TA-DPQP/*p*-PDA (#79) and TA-PTP/*p*-PDA (#69) systems were most suitable for reducing *C*_i_ and, as a result, *W*_A_ and CHE, without any concerns of flame retardancy deterioration.

### 3.6. Performance Balance of Typical PEsIs Examined Herein

From the perspective of practicality, one should aim to achieve the best property balance by overcoming the various trade-offs listed in Figure 1. The performance balance of typical PEsIs described herein was visualized using spider charts, which were prepared by ranking the required properties on a scale of 1–5 (Table 16). For example, *T*_g_ ≥ 360 °C, or non-detectable *T*_g_ even by DMA, was given the highest physical heat resistance rank of 5. This rank referred to *s*-BPDA/*p*-PDA (*T*_g_ = 360–370 °C [81]), which has virtually the highest *T*_g_ among common PIs. In contrast, *T*_g_ ≤ 210 °C was given the lowest rank of 1 with reference to a poly(ether imide) derived from a bisphenol A-type TCDA and *m*-PDA (*T*_g_ = 215 °C [82]), which has virtually the lowest *T*_g_ among common PIs. The above span of *T*_g_ values (360–210 °C) was roughly divided into three equal parts and assigned to the intermediate ranks of 4–2.

Other required properties were ranked in a similar manner; CTE ≤ 10 ppm K^−1^ was ranked 5 with reference to *s*-BPDA/*p*-PDA (CTE = 5–15 ppm K^−1^), which has virtually the lowest CTE among common PIs [57,58], and CTE ≥ 70 ppm K^−1^, which is observed for numerous flexible PIs and common polymers [68], was ranked 1.

*ε*_b max_ ≥ 80% was ranked 5 with reference to PMDA/4,4′-ODA (*ε*_b_ ~ 80% [65,66]), which has virtually the highest *ε*_b_ among common PIs, and *ε*_b max_ ≤ 2% or no film-forming ability (with cracks) was ranked 1 with reference to PMDA/*p*-PDA (*ε*_b_ = 0%).

*W*_A_ ≤ 0.1% was ranked 5 with reference to 6FDA/TFMB PI (*W*_A_ = 0.2% [43]), and *W*_A_ ≥ 3.0% was ranked 1 with reference to PMDA/4,4′-diaminobenzanilide (*W*_A_ = 3.4% [21]), which has virtually the highest *W*_A_ among common PIs.

CHE ≤ 2 ppm/%RH was ranked 5 with reference to a typical ultralow-CHE PEsI, TA-44BPHB;*p*-PDA(75);4,4′-ODA(25) (3.3 ppm/%RH [24]), and CHE ≥ 50 ppm/%RH was ranked 1 with reference to the Neopulim^TM^ (L-1000) film (52 ppm/%RH [83]), which has virtually the highest CHE among PIs.

In this study, systems with V-0, V-1, and V-2 compatibility, and V-2-incompatibility, (Table S4) were assigned to ranks 5, 3, 2, and 1, respectively.

**Figure 15:**
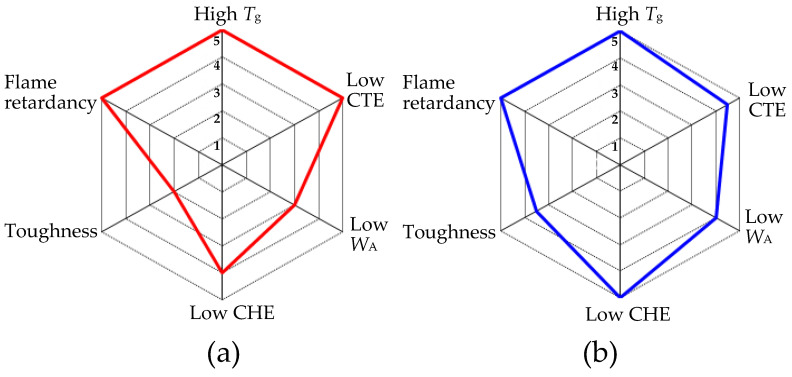
Performance balance based on 5-ranked criteria (Table 16) for typical PEsIs examined in this study: (**a**) TA-26NA/*p*-PDA (#32) and (**b**) TA-DPQP/*p*-PDA (#79).

Figure 15a shows the spider chart for TA-26NA/*p*-PDA (#32), revealing that this PEsI achieved excellent combined properties, except for the insufficient film toughness. However, this toughness is assumed to be difficult to significantly improve via copolymerization with flexible diamines (e.g., 4,4′-ODA) while maintaining the other target properties because even the homo TA-26NA/4,4′-ODA system (#31) did not provide a highly tough film.

On the other hand, an approximately evenly spread spider chart was observed for TA-DPQP/*p*-PDA (#79) [Figure 15b], reflecting its excellent property balance between an extremely high *T*_g_, very low CTE, low *W*_A_, ultralow CHE, sufficiently high *ε*_b_, and excellent flame retardancy (V-0).

## 4. Conclusions

This study focused on improving the flame retardancy and film toughness of PEsIs, which deteriorate upon the structural extension of the repeating units (or monomers) via ester groups for reducing *W*_A_ and CHE.

Initially we explored reactive (bifunctional) flame retardants (modifiers) effective in improving the originally insufficient flame retardancy of PEsIs. An originally highly flammable PEsI with excellent combined properties, i.e., the TA-MHQ/BPTP(60);4,4′-ODA(40) system (#3), was modified via copolymerization using various reactive modifiers at low loadings (5 or 10 mol%).

Naphthalene-containing modifiers (1,4,5,8-NTDA and 2,3,6,7-NTDA) and siloxane-based AP-DPSi were ineffective. Moreover, the systems modified using triazine-containing PDA-TAZ and PDA-PTP with a P=S unit remained V-0-incompatible. However, a certain effect was observed; these systems exhibited mild and slow combustion without the development of a large flame, unlike the pristine system (#3). On the other hand, the introduction of AB-HCAHQ (5 mol%) dramatically improved flame retardancy (V-0 passed), despite its very low *C*_p_ of 0.21 wt%.

To identify the structural factors to realize V-0 in the absence of flame retardants, the structure–property relationship was investigated for a series of modifier-free PEsIs. We initially focused on PEsIs derived from various isomeric TA-NAs. For example, the TA-26NA/*p*-PDA system (#32) exhibited the best property combination, i.e., an extremely high *T*_g_, ultralow CTE, very low CHE, and excellent flame retardancy (V-0), but showed insufficient film toughness. However, copolymerization with 4,4′-ODA was deemed an ineffective strategy for improving toughness because even the homo TA-26NA/4,4′-ODA system (#31) did not provide a highly tough film. Thus, the film toughness of TA-26NA-based systems was concluded to be difficult to significantly improve.

We also examined the structure–property relationships for a series of TA-*p*Ph-based PEsIs and discussed the effects of the *L*_pPh_ and substituents (CH_3_ and phenyl) of TA-*p*Ph on the target properties. The TA-DPQP/*p*-PDA system (#79) achieved the best property combination, i.e., an extremely high *T*_g_, very low CTE, low *W*_A_, ultralow CHE, sufficiently high *ε*_b_, and excellent flame retardancy (V-0). A rationalization of the significantly improved toughness and flame retardancy of this system was proposed. Thus, novel high-temperature dielectric materials suitable for FPC applications were obtained.

## Figures and Tables

**Table 1:** The difficulty in simultaneously achieving the required properties without using fluorine-containing monomers: (●) relatively easy, (■) moderate, (♦) somewhat difficult or difficult, and (⁕) very difficult.

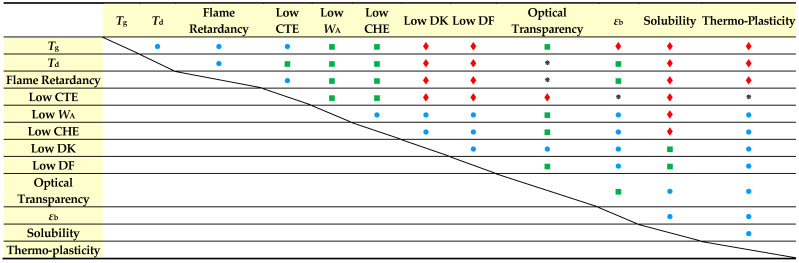

**Table 2:** Impact of incorporation of ester groups to PI main chains on flame retardancy (UL-94, V-0).

No.	Chain Structure	UL-94, V-0 (*d*, μm)
1	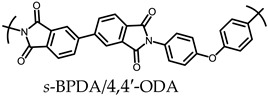	Passed (30)
2	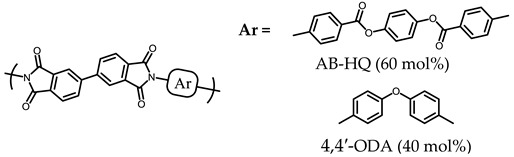	Failed (24)

**Table 3:** Effects of modifiers (shown in bold in this table) on flame retardancy of a PEsI copolymer derived from TA-MHQ with BPTP and 4,4′-ODA. The structures of the PEsI and modifiers, and their abbreviations are shown at the bottom of this table.

No.	TCDA (mol%)	Diamine (mol%)	*η*_red_ PAA (dL g^−1^)	*T*_g_ (°C)	CTE (ppm K^−1^)	Δ*n*_th_	*E* (GPa)	*ε*_b_ av/max (%)	*σ*_b_ (GPa)	*T*_d_^5^ in N_2_ (°C)	*T*_d_^5^ in Air (°C)	*W*_A_ (%)	CHE (ppm/%RH)	UL-94 V-0 (*d*, μm)
3	TA-MHQ	BPTP (60) 4,4′-ODA (40)	3.76	390 ^a^ 200 ^b^	12.3	0.137	4.32	26.8 41.6	0.28	461	444	0.52	5.0	Failed (25)
4*x*	TA-MHQ (95) **1,4,5,8-NTDA (5)**	BPTP (60) 4,4′-ODA (40)	1.81	386 ^c^	10.8	0.170	3.76	25.8 38.2	0.24	444	444	0.59	---	Failed (25)
4*y*	TA-MHQ (90) **1,4,5,8-NTDA (10)**	BPTP (60) 4,4′-ODA (40)	1.06	382 ^c^	31.8	0.112	3.94	14.9 27.0	0.21	445	442	0.62	---	Failed (20)
5*x*	TA-MHQ (95) **2,3,6,7-NTDA (5)**	BPTP (60) 4,4′-ODA (40)	3.96	397 ^a^ 381 ^c^	14.7	0.170	5.30	25.4 45.0	0.32	447	447	0.57	---	Failed (22)
5*y*	TA-MHQ (90) **2,3,6,7-NTDA (10)**	BPTP (60) 4,4′-ODA (40)	4.85	385 ^c^	12.5	0.173	4.30	36.0 55.4	0.31	448	452	0.56	---	Failed (23)
6*x*	TA-MHQ	BPTP (55) 4,4′-ODA (40) **AP-DPSi (5)**	5.40	386 ^c^	11.9	0.149	3.95	30.6 48.4	0.27	462	445	0.54	---	Failed (24)
6*y*	TA-MHQ	BPTP (50) 4,4′-ODA (40) **AP-DPSi (10)**	4.46	388 ^c^	13.4	0.155	3.53	26.3 39.1	0.25	463	441	0.59	---	Failed (24)
7*x*	TA-MHQ	BPTP (55) 4,4′-ODA (40) **PDA-TAZ (5)**	5.07	381 ^a^	17.4	---	4.09	22.4 47.3	0.25	460	433	0.51	---	Failed (23)
7*y*	TA-MHQ	BPTP (50) 4,4′-ODA (40) **PDA-TAZ (10)**	6.30	373 ^a^	15.3	---	4.41	17.2 27.3	0.23	455	429	0.62	3.7	Failed (22)
8*x*	TA-MHQ	BPTP (55) 4,4′-ODA (40) **PDA-PTP (5)**	7.04	382 ^c^	14.8	0.166	4.41	23.7 40.1	0.26	482	450	0.73	5.2	Failed (23)
8*y*	TA-MHQ	BPTP (50) 4,4′-ODA (40) **PDA-PTP (10)**	5.19	379 ^c^	15.9	0.159	4.69	35.4 47.4	0.32	477	454	0.87	6.1	Failed (22)
9*x*	TA-MHQ	BPTP (55) 4,4′-ODA (40) **AB-HCAHQ (5)**	3.56	382 ^a^	15.7	0.139	4.08	24.6 37.3	0.30	450	439	0.54	5.5	Passed (24)
9*y*	TA-MHQ	BPTP (50) 4,4′-ODA (40) **AB-HCAHQ (10)**	2.61	378 ^a^	18.5	0.139	4.66	17.8 32.7	0.24	452	442	0.56	6.1	Passed (25)
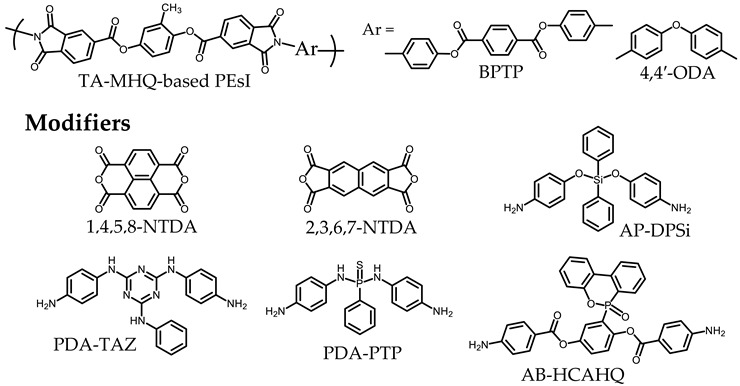														

^a^ *T*_g_ determined by DMA. ^b^ A weak *β*-transition observed by DMA. ^c^ *T*_g_ determined by TMA.

**Table 4:** Film properties of PEsIs derived from TA-14NA with aromatic diamines. The structures of TA-14NA and its analog (TA-HQ) are shown at the bottom of this table.

No.	Diamine	*η*_red_ PAA (dL g^−1^)	*T*_g_ (°C)	CTE (ppm K^−1^)	*E* (GPa)	*ε*_b_ av/max (%)	*σ*_b_ (GPa)	*T*_d_^5^ in N_2_ (°C)	*T*_d_^5^ in Air (°C)	*W*_A_ (%)	CHE (ppm/%RH)
10	4,4′-ODA	3.00	378 (320) ^b^	48.2 (51.2) ^b^	--- ^a^	--- ^a^	--- ^a^	439	428	1.18	10.8
11	*p*-PDA	3.11	423 (385) ^c,d^	5.0 (3.2) ^c^	6.67	2.5 3.3	0.131	457 (481) ^c^	445 (463) ^c^	1.32	4.7
12	*m*-TOL	4.07	354	15.6	5.46	3.3 5.4	0.160	437	426	1.06	2.5
13	*o*-TOL	3.07	381	17.9	5.84	3.9 4.8	0.179	446	425	1.17	4.7
14	APAB	1.24	384	10.0	--- ^a^	--- ^a^	--- ^a^	455	444	0.67	1.8
15	M-APAB	1.10	359	7.0	5.66	3.1 5.0	0.162	444	428	0.82	---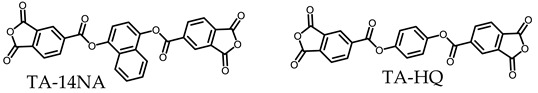											

^a^ Data not available for film brittleness. ^b^ Data for the TA-HQ/4,4′-ODA system [21]. ^c^ Data for the TA-HQ/*p*-PDA system [21]. ^d^ *T*_g_ was barely observed at 385 °C although it was very unclear.

**Table 5:** Film properties of PEsIs derived from TA-15NA with aromatic diamines. The structure of TA-15NA is shown at the bottom of this table.

No.	Diamine	*η*_red_ PAA (dL g^−1^)	*T*_g_ (°C)	CTE (ppm K^−1^)	*E* (GPa)	*ε*_b_ av/max (%)	*σ*_b_ (GPa)	*T*_d_^5^ in N_2_ (°C)	*T*_d_^5^ in Air (°C)	*W*_A_ (%)	CHE (ppm/%RH)
16	4,4′-ODA	0.83	336	51.8	3.77	3.4 5.3	0.113	447	447	0.77	9.6
17	*p*-PDA	0.94	362	21.4	4.40	3.5 4.6	0.117	464	459	1.28	6.6
18	*m*-TOL	1.05	376	23.8	4.13	3.0 4.4	0.125	444	451	1.31	4.7
19	*o*-TOL	1.36	385	19.1	3.46	3.7 5.3	0.108	444	440	0.67	4.5
20	APAB	0.69	383	22.5	3.86	6.1 11.5	0.157	464	458	0.68	2.3
21	M-APAB	0.83	388	15.4	3.93	3.7 5.1	0.109	447	431	0.60	1.9
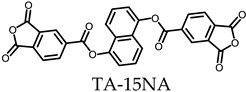											

**Table 6:** Film properties of PEsIs derived from TA-16NA with aromatic diamines. The structure of TA-16NA is shown at the bottom of this table.

No.	Diamine	*η*_red_ PAA (dL g^−1^)	CTE (ppm K^−1^)	*E* (GPa)	*ε*_b_ av/max (%)	*σ*_b_ (GPa)	*T*_d_^5^ in N_2_ (°C)	*T*_d_^5^ in Air (°C)	*W*_A_ (%)	CHE (ppm/%RH)
22	4,4′-ODA	1.17	60.8	2.00	10.0 14.7	0.109	458	461	0.80	---
23	*p*-PDA	1.21	50.9	3.04	6.9 10.7	0.134	462	468	1.48	20.9
24	APAB	1.40	52.2	2.37	9.5 16.5	---	459	459	0.86	15.3
25	M-APAB	0.65	52.1	2.59	6.4 8.7	0.108	458	438	1.48	13.6
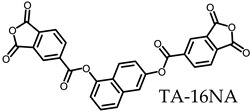										

**Table 7:** Film properties of PEsIs derived from TA-27NA with various aromatic diamines. The structure of TA-27NA is shown at the bottom of this table.

No.	Diamine	*η*_red_ PAA (dL g^−1^)	*T*_g_ (°C)	CTE (ppm K^−1^)	*E* (GPa)	*ε*_b_ av/max (%)	*σ*_b_ (GPa)	*T*_d_^5^ in N_2_ (°C)	*T*_d_^5^ in Air (°C)	*W*_A_ (%)	CHE (ppm/%RH)
26	4,4′-ODA	1.05	381 ^a^ 244 ^b^	50.4	3.05	4.1 5.3	0.096	447	447	0.79	4.1
27	*p*-PDA	3.26	404 ^a^	22.7	--- ^c^	--- ^c^	--- ^c^	464	453	1.32	16.1
28	*o*-TOL	3.34	300 ^a^ 241 ^b^	25.6	3.61	21.9 39.9	0.188	451	441	1.08	4.1
29	APAB	3.02	344 ^a^	29.1	5.31	3.3 5.3	0.177	453	448	1.05	14.5
30	M-APAB	1.80	352 ^a^	28.7	4.36	4.2 5.7	0.130	442	431	1.03	8.1
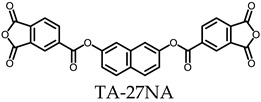											

^a^ *T*_g_ determined by DMA. ^b^ *T*_β_ determined by DMA. ^c^ Data not available for film brittleness.

**Table 8:** Film properties of PEsIs derived from TA-26NA with various aromatic diamines. The structure of TA-26NA is shown at the bottom of this table.

No.	Diamine	*η*_red_ PAA (dL g^−1^)	*T*_g_ (°C)	CTE (ppm K^−1^)	*E* (GPa)	*ε*_b_ av/max (%)	*σ*_b_ (GPa)	*T*_d_^5^ in N_2_ (°C)	*T*_d_^5^ in Air (°C)	*W*_A_ (%)	CHE (ppm/%RH)
31	4,4′-ODA	2.41	361 ^a^ 208 ^b^	41.2	3.15	8.3 11.5	0.142	475	468	0.54	2.1
32	*p*-PDA	2.74	418 ^a^	2.9	5.96	3.6 5.5	0.191	499	489	1.03	4.1
33	*m*-TOL	3.81	390 ^a^ 212 ^b^	29.4	6.05	4.0 5.6	0.188	450	461	0.84	1.8
34	*o*-TOL	3.42	413 ^a^	15.6	4.68	4.5 7.4	0.184	471	445	1.23	1.0
35	APAB	1.00	374 ^a^	14.6	4.71	5.5 7.8	0.183	482	480	0.89	4.7
36	M-APAB	0.89	398 ^a^	13.0	5.26	4.4 7.5	0.175	464	439	0.76	2.5
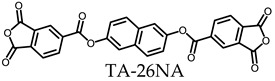											

^a^ *T*_g_ determined by DMA. ^b^ *T*_β_ determined by DMA.

**Table 9:** Film properties of PEsIs derived from TA-44BP with various aromatic diamines. The structure of TA-44BP is shown at the bottom of this table.

No.	Diamine	*η*_red_ PAA (dL g^−1^)	*T*_g_ (°C)	CTE (ppm K^−1^)	*E* (GPa)	*ε*_b_ av/max (%)	*σ*_b_ (GPa)	*T*_d_^5^ in N_2_ (°C)	*T*_d_^5^ in Air (°C)	*W*_A_ (%)	CHE (ppm/%RH)
37	4,4′-ODA	5.48	376 ^c^ 219 ^d^	47.7	2.56	32.6 51.5	0.172	493	488	0.30	0.23
38	*p*-PDA	13.6	ND ^a^	4.3	4.85	6.9 9.6	0.223	511	501	0.91	5.5
39	*m*-TOL	7.90	369 ^b^	22.5	4.95	5.7 6.2	0.194	482	474	1.08	---
40	*o*-TOL	7.87	405 ^b^	11.1	7.25	5.4 6.7	0.268	472	446	0.80	---
41	APAB	1.53	409 ^b^	11.5	5.07	8.7 9.9	0.210	497	493	0.57	---
42	M-APAB	1.12	372 ^b^	10.8	5.83	6.5 8.2	0.210	465	440	0.57	---
43	TFMB	1.69	405 ^c^	16.9	6.80	7.1 11.6	0.180	508	475	0.33	---
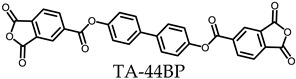											

^a^ *T*_g_ not detected by DMA. ^b^ *T*_g_ determined by DMA. ^c^ *T*_g_ determined by TMA. ^d^ *T*_β_ determined by DMA.

**Table 10:** Film properties of PEsIs derived from TA-DMBP with various diamines. The structure of TA-DMBP is shown at the bottom of this table.

No.	Diamine	*η*_red_ PAA (dL g^−1^)	*T*_g_ (°C)	CTE (ppm K^−1^)	*E* (GPa)	*ε*_b_ av/max (%)	*σ*_b_ (GPa)	*T*_d_^5^ in N_2_ (°C)	*T*_d_^5^ in Air (°C)	*W*_A_ (%)	CHE (ppm/%RH)
44	4,4′-ODA	4.10	347 ^a^ 188 ^b^	55.6	3.27	16.0 23.5	0.175	451	425	0.47	---
45	*p*-PDA	1.65	~420 ^a^	6.7	7.98	6.4 9.3	0.317	454	430	1.07	3.2
46	*m*-TOL	5.72	364 ^a^ 185 ^b^	21.4	5.99	3.9 4.9	0.186	447	411	0.61	---
47	*o*-TOL	5.36	395 ^a^	13.0	7.62	5.5 7.1	0.289	462	441	0.15	---
48	APAB	1.84	365 ^a^	5.4	5.52	4.8 6.4	0.213	455	436	0.34	
49	M-APAB	1.69	351 ^a^	15.9	8.24	6.0 7.5	0.316	453	420	0.62	---
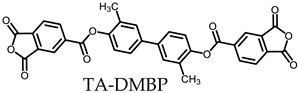											

^a^ *T*_g_ determined by DMA. ^b^ *T*_β_ determined by DMA.

**Table 11:** Film properties of PEsIs derived from TA-DPBP with various diamines. The structure of TA-DPBP is shown at the bottom of this table.

No.	Diamine	*η*_red_ PAA (dL g^−1^)	*T*_g_ (°C)	CTE (ppm K^−1^)	*E* (GPa)	*ε*_b_ av/max (%)	*σ*_b_ (GPa)	*T*_d_^5^ in N_2_ (°C)	*T*_d_^5^ in Air (°C)	*W*_A_ (%)	CHE (ppm/%RH)
50	4,4′-ODA	1.62	222 ^a^	60.1	2.73	11.4 17.8	0.140	471	459	0.23	---
51	3,4′-ODA	0.59	207 ^a^	64.8	2.44	26.2 29.8	0.110	474	466	0.30	---
52	BAPS	1.00	228 ^a^	61.3	2.47	7.5 10.6	0.116	474	472	0.39	---
53	*p*-PDA	1.57	347 ^b^ 235 ^d^	21.7	4.43	11.6 14.0	0.237	481	470	0.65	3.9
54	*m*-TOL	1.78	349 ^b^ 205 ^d^	24.4	3.83	6.1 6.9	0.170	449	468	0.45	---
55	*o*-TOL	0.63	331 ^b^ 207 ^d^	28.0	3.94	4.6 5.4	0.168	465	456	0.39	---
56	APAB	1.14	342 ^c^ 213 ^d^	24.4	3.80	9.9 13.1	0.179	465	463	0.43	---
57	M-APAB	0.76	297 ^c^ 199 ^d^	26.9 ^e^	4.36	6.7 9.8	0.172	462	434	0.50	---
58	TFMB	0.64	197 ^a^	52.5	3.46	4.3 4.9	0.125	482	471	---	---
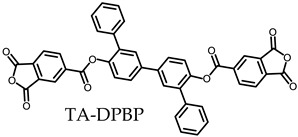											

^a^ A distinct thermal transition temperature (*T*_β_) determined by DMA. ^b^ *T*_g_ determined by DMA. ^c^ *T*_g_ determined by TMA. ^d^ *T*_β_ determined by DMA. ^e^ Data averaged in the range of 100–150 °C.

**Table 12:** Film properties of PEsIs derived from TA-MTP with various diamines. The structure of TA-MTP is shown at the bottom of this table.

No.	Diamine	*η*_red_ PAA (dL g^−1^)	*T*_g_ (°C)	CTE (ppm K^−1^)	*E* (GPa)	*ε*_b_ av/max (%)	*σ*_b_ (GPa)	*T*_d_^5^ in N_2_ (°C)	*T*_d_^5^ in Air (°C)	*W*_A_ (%)	CHE (ppm/%RH)
59	4,4′-ODA	1.26	377 ^a^ 193 ^b^	56.2	2.72	21.8 25.7	0.189	472	466	0.71	---
60	*p*-PDA	1.49	422 ^a^	5.5	7.61	5.3 6.3	0.299	479	461	0.93	3.4
61	*m*-TOL	1.81	325 ^a^	20.7	5.62	14.4 18.2	0.300	467	469	0.69	---
62	*o*-TOL	2.33	407 ^a^	19.1	6.51	5.4 6.0	0.248	472	462	0.58	0.8
63	APAB	0.84	373 ^a^	14.1	5.87	5.3 6.3	0.193	469	460	0.75	---
64	M-APAB	0.73	373 ^a^ 199 ^b^	20.5	5.26	4.7 5.3	0.177	467	447	1.00	---
65	TFMB	1.19	350 ^a^	30.9	4.76	13.7 26.1	0.150	473	459	0.26	---
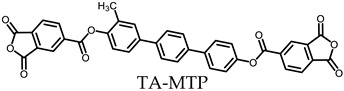											

^a^ *T*_g_ determined by DMA. ^b^ *T*_β_ determined by DMA.

**Table 13:** Film properties of PEsIs derived from TA-PTP with various diamines. The structure of TA-PTP is shown at the bottom of this table.

No.	Diamine	*η*_red_ PAA (dL g^−1^)	*T*_g_ (°C)	CTE (ppm K^−1^)	*E* (GPa)	*ε*_b_ av/max (%)	*σ*_b_ (GPa)	*T*_d_^5^ in N_2_ (°C)	*T*_d_^5^ in Air (°C)	*W*_A_ (%)	CHE (ppm/%RH)
66	4,4′-ODA	1.22	337 ^a^ 197 ^c^	50.1	3.13	40.5 47.5	0.195	484	482	0.45	---
67	3,4′-ODA	1.09	198 ^b^	28.4	3.68	37.2 52.4	0.161	488	480	0.44	---
68	BAPS	0.95	235 ^b^	45.2	2.53	4.7 6.2	0.106	492	478	0.58	---
69	*p*-PDA	1.84	348 ^a^ 254 ^c^	15.7	4.22	14.3 25.5	0.203	487	484	0.62	3.1
70	*m*-TOL	2.81	371 ^a^ 205 ^c^	22.3	5.65	8.4 12.3	0.223	474	472	0.34	---
71	*o*-TOL	2.52	354 ^a^ 249 ^c^	16.2	4.68	13.4 20.6	0.258	477	473	0.25	1.6
72	APAB	1.03	316 ^a^ 181 ^c^	23.9	4.14	21.6 27.4	0.241	481	477	0.33	---
73	M-APAB	0.99	309 ^a^ 196 ^c^	38.2	3.29	19.7 33.5	0.195	466	461	0.42	---
74	TFMB	1.32	205 ^b^	49.1	3.16	64.5 98.0	0.162	498	494	0.15	---
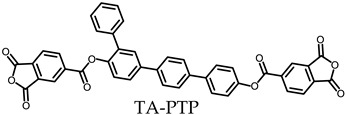											

^a^ *T*_g_ determined by DMA. ^b^ A distinct thermal transition temperature (*T*_β_) determined by TMA. ^c^ *T*_β_ determined by DMA.

**Table 14:** Film properties of PEsIs derived from TA-DMQP with various diamines. The structure of TA-DMQP is shown at the bottom of this table.

No.	Diamine	*η*_red_ PAA (dL g^−1^)	*T*_g_ ^a^ (°C)	CTE (ppm K^−1^)	*E* (GPa)	*ε*_b_ av/max (%)	*σ*_b_ (GPa)	*T*_d_^5^ in N_2_ (°C)	*T*_d_^5^ in Air (°C)	*W*_A_ (%)	CHE (ppm/%RH)
75	4,4′-ODA	1.52	372	46.7	3.26	31.9 36.5	0.203	474	472	0.47	4.2
76	*p*-PDA (70) 4,4′-ODA (30)	1.26	392	10.7	5.21	8.9 11.5	0.207	481	456	0.51	---
77	APAB (70) 4,4′-ODA (30)	0.66	378	29.1	4.40	10.0 17.7	0.166	483	448	0.26	---
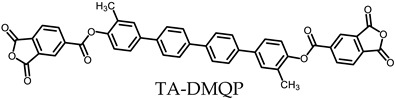											

^a^ *T*_g_ determined by DMA.

**Table 15:** Film properties of PEsIs derived from TA-DPQP with various diamines. The structure of TA-DPQP is shown at the bottom of this table.

No.	TCDA	Diamine	*η*_red_ PAA (dL g^−1^)	*T*_g_ (°C)	CTE (ppm K^−1^)	*E* (GPa)	*ε*_b_ av/max (%)	*σ*_b_ (GPa)	*T*_d_^5^ in N_2_ (°C)	*T*_d_^5^ in air (°C)	*W*_A_ (%)	CHE (ppm/%RH)
78	TA-DPQP	4,4′-ODA	3.89	201 ^a^	41.6	2.56	32.6 51.5	0.172	482	477	0.35	---
79	ibid	*p*-PDA	5.67	412 ^c^ 220 ^d^	12.7	3.22	31.3 36.2	0.207	501	467	0.38	2.0
80	ibid	*m*-TOL	5.83	311 ^c^ 210 ^d^	28.6	5.34	13.0 15.3	0.258	484	456	0.33	---
81	ibid	*o*-TOL	5.72	364 ^b^ 244 ^d^	15.2	5.40	4.7 5.8	0.196	470	444	--- ^g^	---
82	ibid	APAB	1.40	329 ^c^ 224 ^d^	21.8	3.76	11.6 15.5	0.199	495	480	0.46	---
83	ibid	M-APAB	1.11	296 ^c^ 200 ^d^	22.1	5.05	9.8 11.2	0.268	473	454	0.41	---
84 ^e^	TA-DPQP (70) TA-44BP (30)	*p*-PDA	3.04	327 ^c^ 224 ^d^	13.0	4.08	17.8 22.0	0.258	494	487	0.55	2.6
85 ^f^	ibid	ibid	4.24	325 ^c^ 221 ^d^	15.1	4.03	17.0 29.1	0.211	495	490	0.40	1.3
86 ^e^	TA-DPQP (50) TA-44BP (50)	ibid	3.19	417 ^b^ 225 ^d^	11.3	4.41	12.8 17.7	0.227	498	494	0.54	1.4
87 ^e^	ibid	*p*-PDA (80) 4,4′-ODA (20)	3.26	361 ^b^ 223 ^d^	14.5	3.25	8.5 13.8	0.166	495	488	0.41	1.8
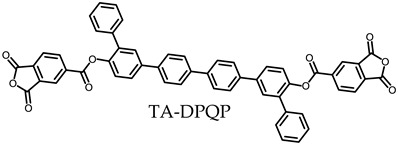												

^a^ A distinct thermal transition temperature (*T*_β_) determined by TMA. ^b^ *T*_g_ determined by DMA. ^c^ *T*_g_ determined by TMA. ^d^ *T*_β_ determined by DMA. ^e^ Random copolymerization by simultaneous addition of two TCDAs to the diamine solution. ^f^ Sequence-controlled copolymerization by first addition of TA-44BP to the diamine solution (+stirring for 2 h) and second addition of TA-DPQP. ^g^ It was difficult to accurately determine *W*_A_ owing to unknown data dispersion only for this sample.

**Table 16:** Criteria for estimating the performance balance of PEsIs for use in the dielectric substrates of high-performance FPCs.

Properties	Parameters	Relative Rank				
		1	2	3	4	5
Physical heat resistance	*T*_g_ (°C)	≤210	220–250	260–290	300–330	≥360 or ND ^a^
Thermal dimensional stability	CTE (ppm K^−1^)	≥70	60–50	45–35	30–20	≤10
Low water uptake	*W*_A_ (%)	≥3.0	2.5–2.0	1.5–1.0	0.6–0.3	≤0.1
Hygroscopic dimensional stability	CHE (ppm/%RH)	≥50	30–20	15–10	8–4	≤2
Film toughness	*ε*_b max_ (%)	No film-forming ability or ≤2	5–10	20–30	40–60	≥80
Flame retardancy	UL-94V	V-2 failed	V-2 passed	V-1 passed	---	V-0 passed

^a^ Non-detected *T*_g_ up to 450 °C on DMA.

## Data Availability

The data supporting this study are available within the article and in the Supplementary Materials.

## Supplementary Materials

The following supporting information can be downloaded at: https://www.mdpi.com/article/10.3390/polym16141967/s1, Table S1: Abbreviations, commercial sources, and melting points of the raw materials used in this study. Table S2: The solvents, melting points, and analytical results of the ester-linked monomers synthesized in this study and related monomers. Table S3: Abbreviations, commercial sources, vacuum-drying conditions before use, and melting points of the common monomers used in this study. Table S4: Rating of UL-94 vertical burning tests. Figure S1: Schematic illustration of UV-94 vertical burning tests (a) and typical appearance of the specimens of V-0-compatible systems after the tests (b). Figure S2: Classification of flame retardants, their typical compounds, and expected effects. BPBrPE = 1,2-bis(2,3,4,5,6-pentabromophenyl)ethane, TBrBPA = tetrabromobisphenol A, BHPPO = *n*-butyl-bis(3-hydroxypropyl)phosphine oxide, DVP = diphenyl vinylphosphonate, and HCA-HQ = 10-(2,5-dihydroxyphenyl)-9,10-dihydro-9-oxa-10-phosphaphenanthrene-10-oxide. Figure S3: Schematic illustrations representing the impact of in-plane chain orientation on CHE: (a) films with high in-plane orientation (=low CTE) and (b) those with low in-plane orientation (=high CTE). Figure S4: DMA curves of PEsIs: (a) TA-44BP/*p*-PDA and (b) TA-DMBP/*p*-PDA systems. Figure S5: DMA curves of the TA-DPBP/*p*-PDA films prepared under different thermal conditions: (a) 250 °C/1 h + 300 °C/1 h under vacuum and (b) additional annealing at 400 °C/1 h under vacuum. Figure S6: Schematic drawings for the chain pull-out from a group of entangled chains without stress concentration in systems with sufficient entanglement (1) and poor entanglement due to rigid/linear main-chain structures (2) and insufficient molecular weights (3) and typical stress–strain curves for tough (a) and brittle PI films (b).
