# Supplementary material for: Poly(ester imide)s with Low Linear Coefficients of Thermal Expansion and Low Water Uptake (VIII): Structure–Flame Retardancy Relationship
Source: Polymers (Basel). 2024 Jul 10;16(14):1967. doi: 10.3390/polym16141967 (PMC11280571; doi:10.3390/polym16141967)
Supplement: Supplementary file 1 [file polymers-16-01967-s001.zip › polymers-3040596-supplementary.pdf]

Supplementary Materials

Table S1. Abbreviations, commercial sources, and melting points of the raw materials used in this study.

Raw materials	Source	Melting point (ºC)
Hydroquinone (HQ)	Wako Chemical	173 [a]
Resorcinol (RC)	Tokyo Chemical Industry (TCI)	111 [b]
Methylhydroquinone (M-HQ)	TCI	126 [b]
Methoxyhydroquinone (MeO-HQ)	TCI	89 [b]
1,4-Dihydroxynaphthalene (14DHNA)	TCI	190 [b]
1,5-Dihydroxynaphthalene (15DHNA)	Wako Chemical	260 [b] (dec.)
1,6-Dihydroxynaphthalene (16DHNA)	Wako Chemical	137 [b]
2,6-Dihydroxynaphthalene (26DHNA)	Wako Chemical	222 [b]
2,7-Dihydroxynaphthalene (27DHNA)	Wako Chemical	180 [b] (dec.)
4,4′-Biphenol (44BP)	TCI	286 [a]
4,4′-Dihydroxy-3,3′-dimethylbiphenyl (33DM-44BP)	Honshu Chemical Industry	164 [a]
4,4′-Dihydroxy-3,3′-diphenylbiphenyl (33DP-44BP)	Honshu Chemical Industry	149 [a]
4,4″-Dihdroxy-3-methyl-*p*-terphenyl (3M-44DHTP)	Honshu Chemical Industry	261 [a]
4,4″-Dihdroxy-3-phenyl-*p*-terphenyl (3P-44DHTP)	Honshu Chemical Industry	196 [a]
4,4‴-Dihdroxy-3,3‴-dimethyl-*p*-quaterphenyl (33DM-44DHQP)	Honshu Chemical Industry	297 [a]
4,4‴-Dihdroxy-3,3‴-diphenyl-*p*-quaterphenyl (33DP-44DHQP)	Honshu Chemical Industry	254 [a]
Trimellitic anhydride chloride (TMAC)	TCI	69 [a]
4-Nitrobenzoyl chloride (4-NBC)	TCI	73 [b]
4-Aminophenol (4-AP)	TCI	187 [b]
Cyanuric chloride (CC) [c]	TCI	147 [a]
10-(2,5-Dihydroxyphenyl)-9,10-dihydro-9-oxa-10-phosphaphenanthrene-10-oxide (HCA-HQ)	Sanko	253 [a]
Dichlorodiphenylsilane (DCDPSi)	TCI	---
Phenylthiophosphonic dichloride (PTP-DC)	TCI	---

[a] Data determined from the endothermic peak temperature measured at a heating rate of 5 ºC min^{-1} on DSC.

[b] Data from the safety data sheet.

[c] Recrystallized from chloroform and dried at 60 ºC for 12 h under vacuum before use.

Table S2. The solvents, melting points, and analytical results of the ester-linked monomers synthesized in this study and related monomers.

Ester-linked monomers	Solvents for synthesis	Solvents for recrystallization	Vacuum-drying condition	Melting point [a] (°C)	Molecular formula (F_w, g/mol)	C, H, N (%) (Calcd.) (Found)
TA-HQ [b]	THF	DOX	200 °C/24 h	277	$C_{24}H_{10}O_{10}$ (458.34)	---
TA-RC	THF	Ac_2O/AcOH (1/1, v/v)	160 °C/12 h	207	$C_{24}H_{10}O_{10}$ (458.34)	---
TA-MHQ [c]	THF	DOX/THF (4/1, v/v)	160 °C/24 h	251	$C_{25}H_{12}O_{10}$ (472.36)	---
TA-MeOHQ [c]	THF	DOX	150 °C/12 h	240	$C_{25}H_{12}O_{11}$ (488.36)	---
TA-14NA	THF	Ac_2O/AcOH (1/1, v/v)	160 °C/12 h	297	$C_{28}H_{12}O_{10}$ (508.40)	---
TA-15NA	THF	GBL	160 °C/12 h	309	ibid	---
TA-16NA	THF	GBL	160 °C/12 h	277	ibid	---
TA-26NA	THF	GBL	160 °C/12 h	302	ibid	66.15, 2.38, 0<br>66.01, 2.54, 0
TA-27NA	THF	Ac_2O	160 °C/12 h	234	ibid	---
TA-44BP	DMF	GBL	200 °C/12 h	326	$C_{30}H_{14}O_{10}$ (534.44)	67.42, 2.64, 0<br>67.66, 2.86, 0
TA-DMBP	DMF	DOX	160 °C/12 h	251	$C_{32}H_{18}O_{10}$ (562.49)	---
TA-DPBP	THF	Ac_2O/AcOH (10/1, v/v)	160 °C/12 h	239	$C_{42}H_{22}O_{10}$ (686.63)	---
TA-MTP	THF	GBL	160 °C/12 h	299	$C_{37}H_{20}O_{10}$ (624.56)	71.16, 3.23, 0<br>71.05, 3.36, 0
TA-PTP	THF	GBL/Toluene (1/6, v/v)	160 °C/12 h	251	$C_{42}H_{22}O_{10}$ (686.63)	73.47, 3.23, 0<br>73.49, 3.38, 0
TA-DMQP	DMF	GBL	200 °C/12 h	302	$C_{44}H_{26}O_{10}$ (714.68)	---
TA-DPQP	DMAc	DOX/toluene (2/3, v/v)	160 °C/12 h	278	$C_{54}H_{30}O_{10}$ (838.83)	77.32, 3.60, 0<br>77.26, 3.66, 0

[a] Data determined from the endothermic peak measured at a heating rate of 5 °C min^{-1} on DSC.

[b] Ref. [**21**]

[c] Ref. [**22**]

DOX = 1,4-dioxane, THF = Tetrahydrofuran, Ac_2O = Acetic anhydride, AcOH = Glacial acetic acid, GBL = *γ*-Butyrolactone, DMF = *N*,*N*-Dimethylformamide.

Table S3. Abbreviations, commercial sources, vacuum-drying conditions before use, and melting points of the common monomers used in this study.

Monomers	Source	Vacuum-drying condition	Melting point (°C)
4,4′-Oxydianiline (4,4′-ODA)	Wako Chemical	50 ºC/12 h	191 [a]
3,4′-Oxydianiline (3,4′-ODA)	JFE Chemical	50 ºC/24 h	132 [a]
Bis[4-(4-aminophenoxy)phenyl] sulfone (BAPS)	Wakayama Seika	50 ºC/24 h	196 [a]
p-Phenylenediamine (*p*-PDA)	Wako Chemical	30 ºC/12 h	141 [a]
m-Tolidine (*m*-TOL)	Tokyo Chemical Industry (TCI)	50 ºC/24 h	106 [b]
o-Tolidine (*o*-TOL)	TCI	50 ºC/24 h	129 [b]
4-Aminophenyl 4-aminobenzoate (APAB)	Wakayama Seika	50 ºC/12 h	185 [a]
4-Amino-2-methylphenyl 4-aminobenzoate (M-APAB)	Wakayama Seika	50 ºC/12 h	162 [a]
Bis(4-aminophenyl) terephthalate (BPTP)	Wakayama Seika	50 ºC/24 h	238 [a]
2,2′-Bis(trifluoromethyl)benzidine (TFMB)	Wakayama Seika	50 ºC/12 h	184 [a]
1,4,5,8-Naphthaneletetracarboxylic dianhydride (1,4,5,8-NTDA)	Aldrich	120 ºC/24 h	451 [a]
2,3,6,7-Naphthaneletetracarboxylic dianhydride (2,3,6,7-NTDA) [c]	JFE Chemical	120 ºC/24 h	360 [a]

[a] Data determined from the endothermic peak temperature measured at a heating rate of 5 ºC min^{-1} on DSC.

[b] Data from the safety data sheet.

[c] Gift from Sony Chemical & Information Device.

Table S4. Rating of UL-94 vertical burning tests.

Evaluation items	Rating		
	V-0	V-1	V-2
Flaming time for each specimen	≤ 10 s	≤ 30 s	≤ 30 s
Burning up to clamped top edge for each specimen	Not allowed	Not allowed	Not allowed
Generation of non-flaming melt-down fragments	Allowed	Allowed	Allowed
Flaming of cotton sheet by melt-down fragments	Not allowed	Not allowed	Allowed

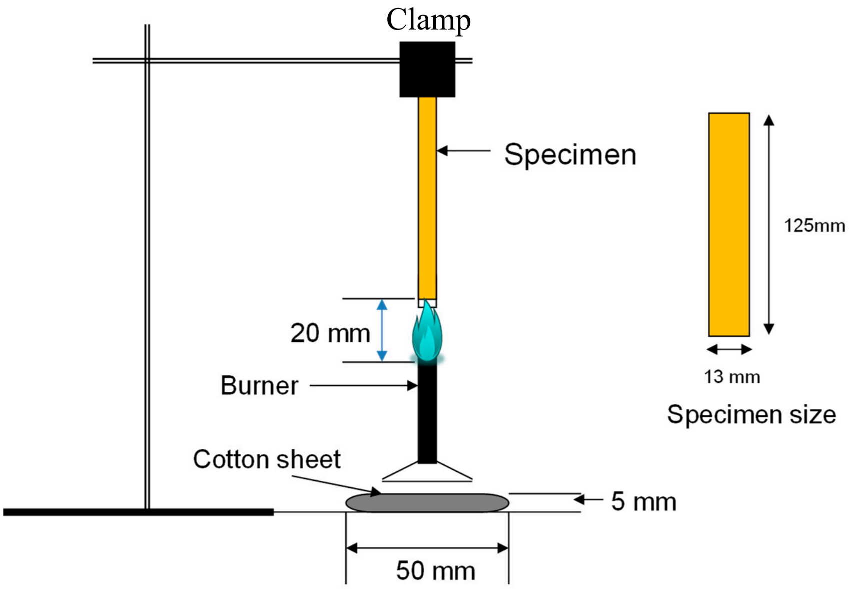

(a)

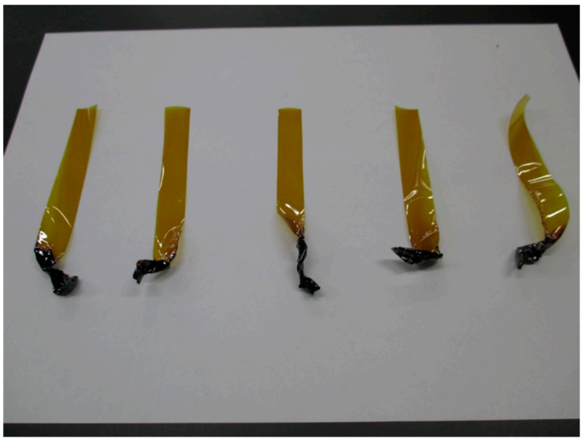

(b)

Figure S1. Schematic illustration of UV-94 vertical burning tests (a) and a typical appearance of the specimens of V-0-compatible systems after the tests (b).

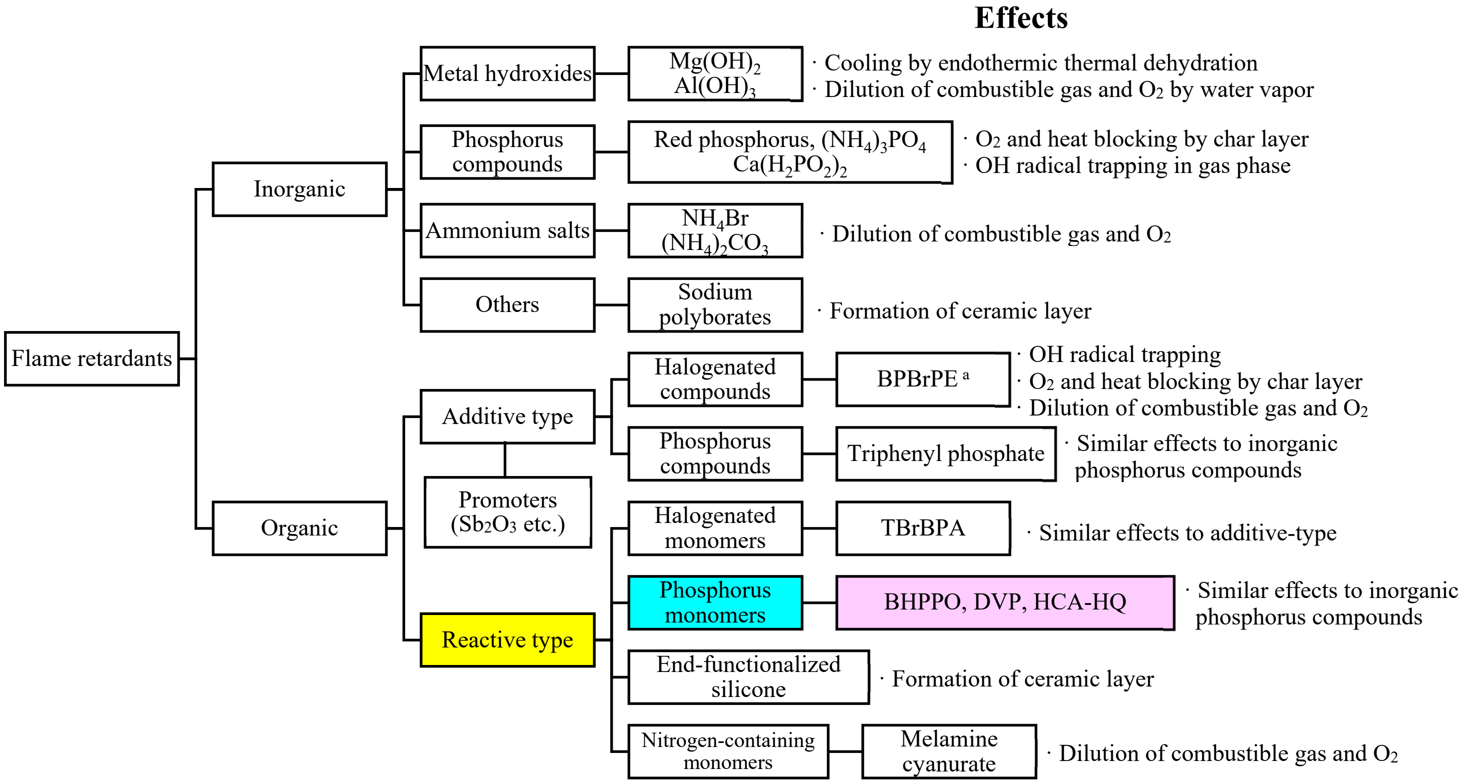

Figure S2. Classification of flame retardants, their typical compounds, and expected effects. BPBrPE = 1,2-bis(2,3,4,5,6-pentabromophenyl)ethane, TBrBPA = tetrabromobisphenol A, BHPPO = *n*-butyl-bis(3-hydroxypropyl)phosphine oxide, DVP = diphenyl vinylphosphonate, and HCA-HQ = 10-(2,5-dihydroxyphenyl)-9,10-dihydro-9-oxa-10-phosphaphenanthrene-10-oxide.

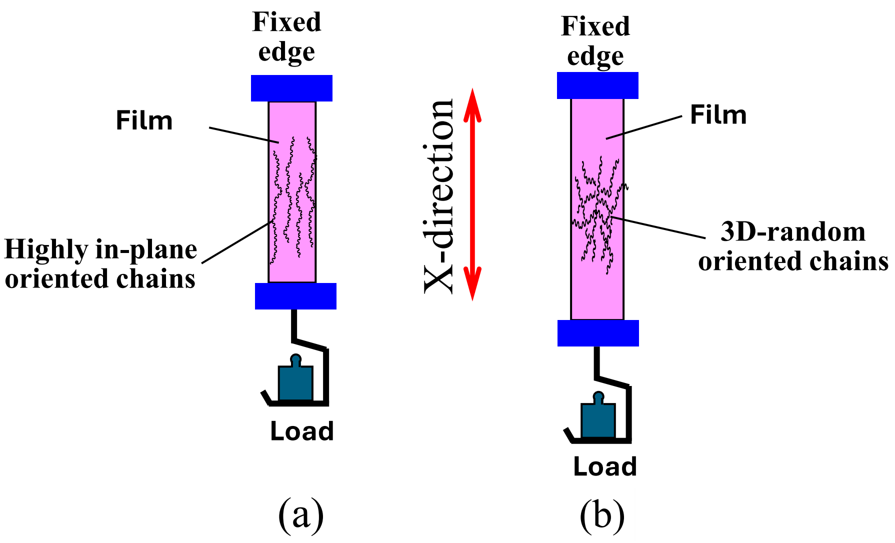

Figure S3. Schematic illustrations representing the impact of in-plane chain orientation on CHE: (a) films with high in-plane orientation (= low CTE) and (b) those with low in-plane orientation (= high CTE).

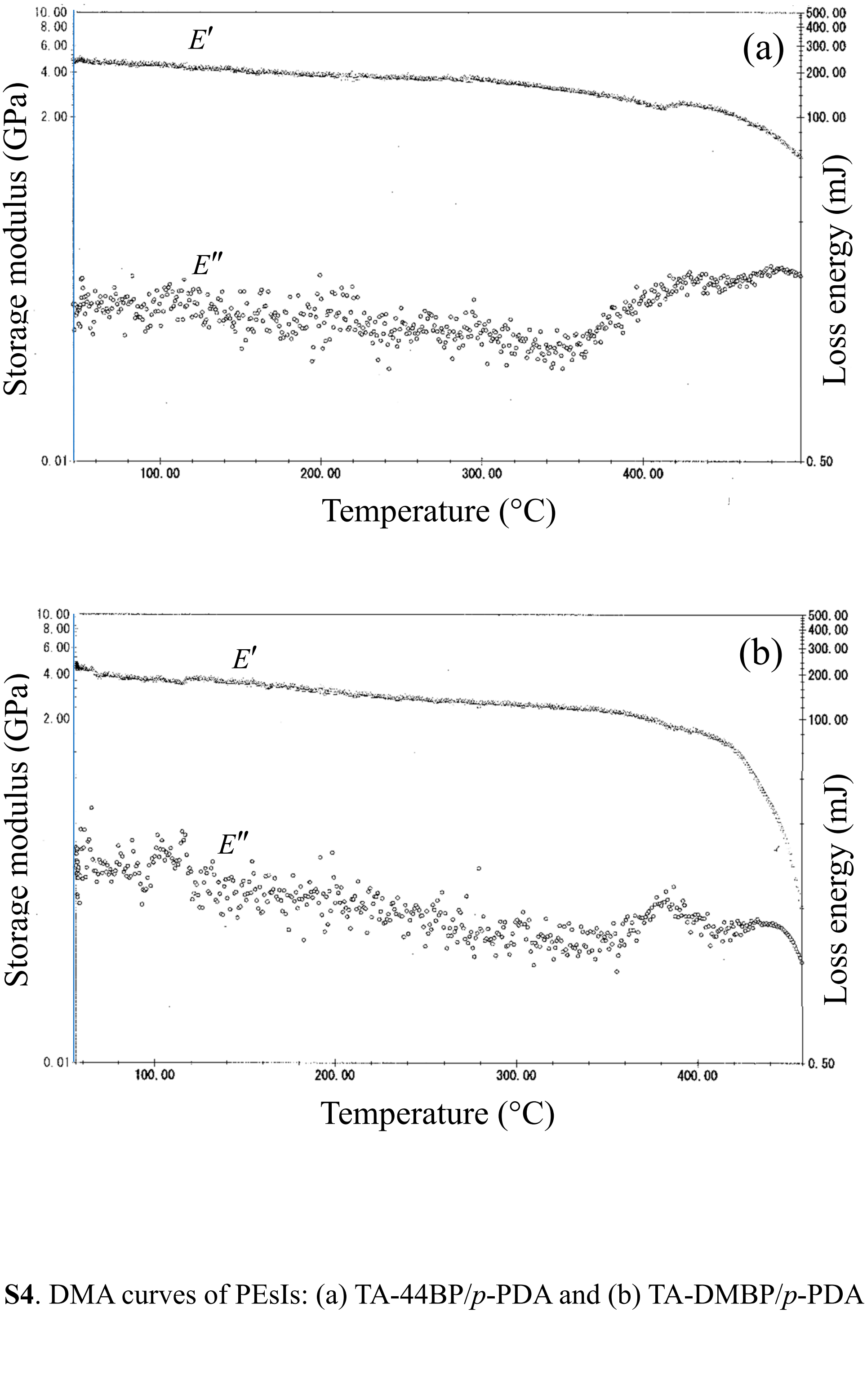

Figure S4. DMA curves of PEsIs: (a) TA-44BP/*p*-PDA and (b) TA-DMBP/*p*-PDA systems.

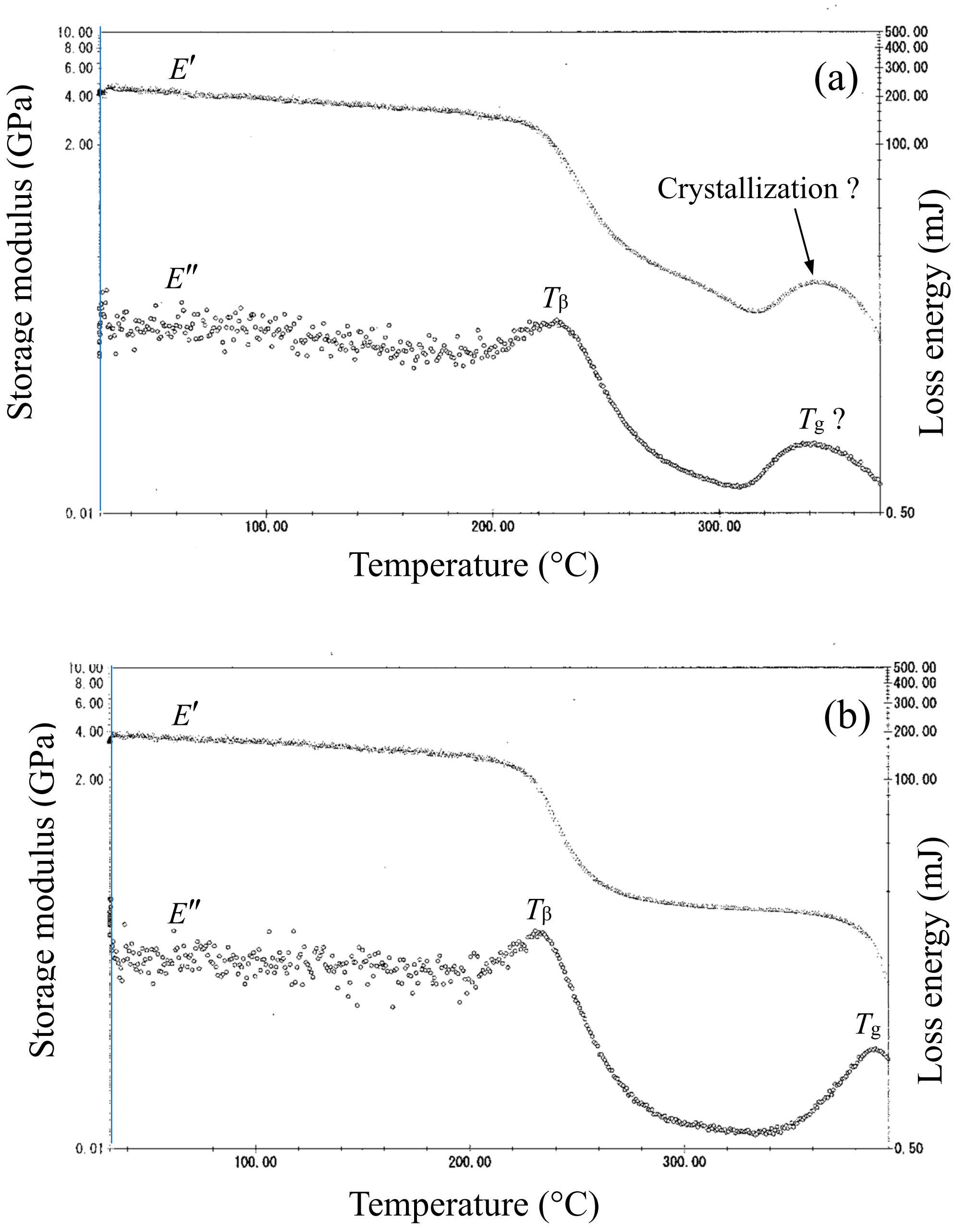

Figure S5. DMA curves of the TA-DPBP/*p*-PDA films prepared under different thermal conditions: (a) 250 °C/1 h + 300 °C/1 h under vacuum and (b) additional annealing at 400 °C/1 h under vacuum.

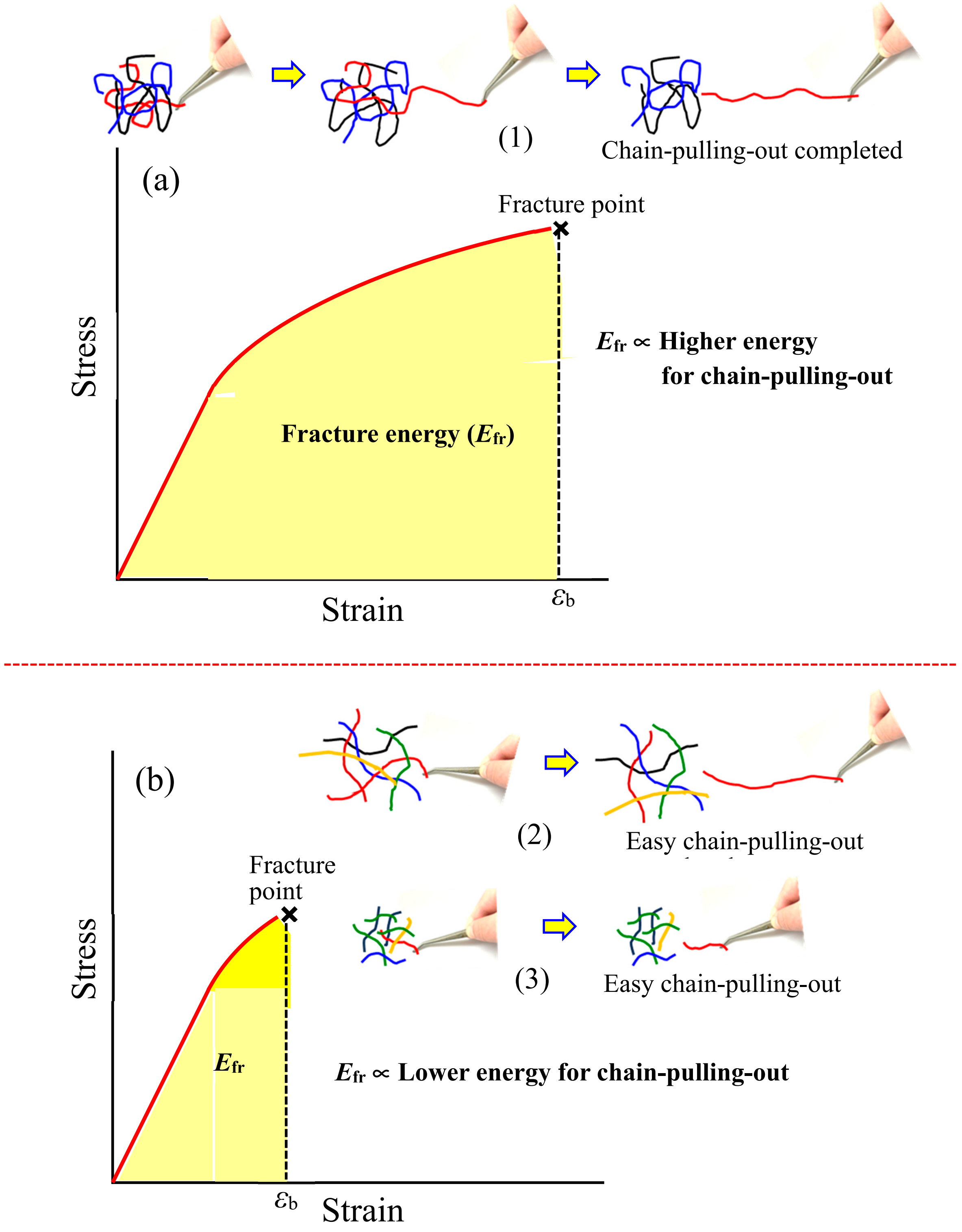

Figure S6. Schematic drawings for the chain pull-out from a group of entangled chains without stress concentration in systems with sufficient entanglement (1) and poor entanglement due to rigid/linear main-chain structures (2) and insufficient molecular weights (3) and typical stress–strain curves for tough (a) and brittle PI films (b).
